# Transformation of Teosinte (*Zea mays* ssp. *parviglumis*) *via* Biolistic Bombardment of Seedling-Derived Callus Tissues

**DOI:** 10.3389/fpls.2021.773419

**Published:** 2021-12-09

**Authors:** Jacob D. Zobrist, Susana Martin-Ortigosa, Keunsub Lee, Mercy K. Azanu, Q Ji, Kan Wang

**Affiliations:** ^1^Department of Agronomy, Iowa State University, Ames, IA, United States; ^2^Crop Bioengineering Center, Iowa State University, Ames, IA, United States; ^3^Interdepartmental Genetics and Genomics Major, Iowa State University, Ames, IA, United States; ^4^Interdepartmental Plant Biology Major, Iowa State University, Ames, IA, United States

**Keywords:** embryogenic callus, gene gun, genetic transformation, growth media, herbicide resistance, mature seed, *Zea parviglumis*

## Abstract

Modern maize exhibits a significantly different phenotype than its wild progenitor teosinte despite many genetic similarities. Of the many subspecies of *Zea mays* identified as teosinte, *Zea mays* ssp. *parviglumis* is the most closely related to domesticated maize. Understanding teosinte genes and their regulations can provide great insights into the maize domestication process and facilitate breeding for future crop improvement. However, a protocol of genetic transformation, which is essential for gene functional analyses, is not available in teosinte. In this study, we report the establishment of a robust callus induction and regeneration protocol using whorl segments of seedlings germinated from mature seeds of *Zea parviglumis*. We also report, for the first time, the production of fertile, transgenic teosinte plants using the particle bombardment. Using herbicide resistance genes such as mutant acetolactate synthase (*Als*) or bialaphos resistance (*bar*) as selectable markers, we achieved an average transformation frequency of 4.17% (percentage of independent transgenic events in total bombarded explants that produced callus). Expression of visual marker genes of red fluorescent protein *tdTomato* and β-glucuronidase (*gus*) could be detected in bombarded callus culture and in T1 and T2 progeny plants. The protocol established in this work provides a major enabling technology for research toward the understanding of this important plant in crop domestication.

## Introduction

Maize is the most important grain crop for both humans and animals. It is widely believed that the ancestor of modern maize (*Zea mays* ssp. *mays*) is the Mesoamerican plant teosinte. There are many different subspecies of *Zea mays* but data from genetic studies have provided strong evidence that the subspecies *Zea mays* ssp. *parviglumis* (hereafter *Zea parviglumis*) is the wild ancestor of domesticated maize (Doebley, 1990; Matsuoka et al., 2002). There are great interests in tracing the ancestry from this ancient grass to the modern crop using genetics, genomics and genome editing technologies. To this end, the ability to achieve genetic transformation of teosinte is essential.

Limited literature exists on teosinte *in vitro* culture. There is no report on protocols for genetic transformation of any teosinte subspecies. Cure and Mott (1978) performed callus culture in *Zea mays* ssp. *mexicana* using growth medium with synthetic auxin 2,4-dichlorophenoxyacetic acid (2,4-D). They were unable to get regenerated shoots on hormone-free media (Cure and Mott, 1978). One group published two manuscripts in 1984, reporting the production of embryogenic callus from field grown seedling leaf pieces (Prioli et al., 1984; Sondahl et al., 1984) or immature embryos (Sondahl et al., 1984) using MS salts containing 2,4-D in *Zea mays* ssp. *diploperennis* (hereafter *Zea diploperennis*). This group was successful in regenerating many fertile plants from these callus cultures by removal of the 2,4-D from the growth media (Prioli et al., 1984; Sondahl et al., 1984). Swedlund and Locy (1988) also reported the establishment of embryogenic callus using both immature embryos and immature leaf tissue of *Zea diploperennis* on MS/sucrose-based growth media containing 2,4-D. These callus cultures were maintained for up to 2years after callus induction was initiated. They were able to regenerate plants from the 2-year-old callus culture on rooting media containing kinetin and examined the cellular and morphological changes. They stated that “regenerated plants did not differ in chromosome numbers or general morphology from the original plants placed in culture” (Swedlund and Locy, 1988). Zale et al. (2008) described *in vitro* multishoot propagation in *Zea diploperennis*. They found that laterally cut shoots collected from the field could be propagated into clonal plants rapidly when placed onto growth medium containing 5μM 6-benzylamino purine (BAP) to induce multishoot formation from meristematic regions (Zale et al., 2008).

The first successful *Zea* transformation was biolistic-mediated maize transformation that was reported in 1990 (Gordon-Kamm et al., 1990). In this landmark work, immature embryos from a hybrid progeny of maize A188×B73 were used first to initiate friable, embryogenic type II callus culture in growth media containing auxin dicamba or 2,4-D. The callus culture was then developed to cell suspension culture in liquid MS medium with the auxin. The suspension culture was bombarded using a plasmid DNA carrying the herbicide bialaphos resistance (*bar*) gene or co-bombarded using two plasmid DNAs, one carrying the *bar* gene and the another carrying β-glucuronidase (*gus*) gene. Transformed calli were selected from the suspension culture containing the herbicide bialaphos. Over 50 herbicide-resistant independent callus events were regenerated and brought to maturity for progeny analysis.

While the first fertile transgenic maize was produced by the biolistic method, the current most popular approach for maize transformation is the *Agrobacterium*-mediated protocol. The target explant for the *Agrobacterium*-mediated method is maize immature embryos, which are from young ears harvested 10–14days after pollination. While maize is naturally recalcitrant to *Agrobacterium tumefaciens*, a wide range of maize genotypes can now be transformed using *Agrobacterium*-mediated method thanks to the development of super binary vectors and morphogenic regulator genes (Ishida et al., 1996; Zhao et al., 2002; Lowe et al., 2016; Masters et al., 2020).

The aim of this work was to develop a regenerable tissue culture system for teosinte *Zea parviglumis*, the hypothetical ancestor of modern maize. More importantly, this work is intended to establish a genetic transformation protocol that can be used to perform genome editing. Because teosinte has small ears with only 5–12 kernels that are protected by a hard casing (Doebley, 2004), it is difficult to obtain large quantities of kernels and use their immature embryos for tissue culture and transformation, as is typically done for maize. On the other hand, maize mature seeds have been used for transformation. Sidorov et al. (2006) demonstrated that an embryogenic callus culture could be initiated from mature seedlings that were germinated on MS-based medium containing 10mg/L auxin picloram and 3mg/L BAP. They described that the callus pieces were typically produced from leaf coleoptilar node area, which contains meristematic cells and axillary buds. The callus culture was transformed using *Agrobacterium*-mediated protocol. Transgenic plants were successfully produced using either neomycin phosphotransferase II or glyphosate as selective agents (Sidorov et al., 2006). Using morphogenic regulators *Baby boom* and *Wuschel* genes, Lowe et al. (2016) showed that maize mature seed or leaf segments from seedlings could be used as explants for transformation.

In this study, we describe a robust callus culture and regeneration system initiated from mature seed-derived leaf explants. Using embryogenic callus culture, we successfully generated the first fertile, transgenic teosinte (*Zea parviglumis*) plants using the particle bombardment. This work marks a major step forward toward the understanding of this important plant through genome editing and genetic modification.

## Materials and Methods

### Plant Material and Media

*Zea mays* ssp. *parviglumis* (teosinte) seeds Ames 21785 and Ames 21789 were obtained from the USDA Agricultural Research Service seed repository.^1^ Bulk amount of *Zea parviglumis* seeds can be purchased from a private retailer Restoration Seeds.^2^ Culture media used in this work are listed in Supplementary Table S1.

### Seed Sterilization and Explant Preparation

**Figure 1:**
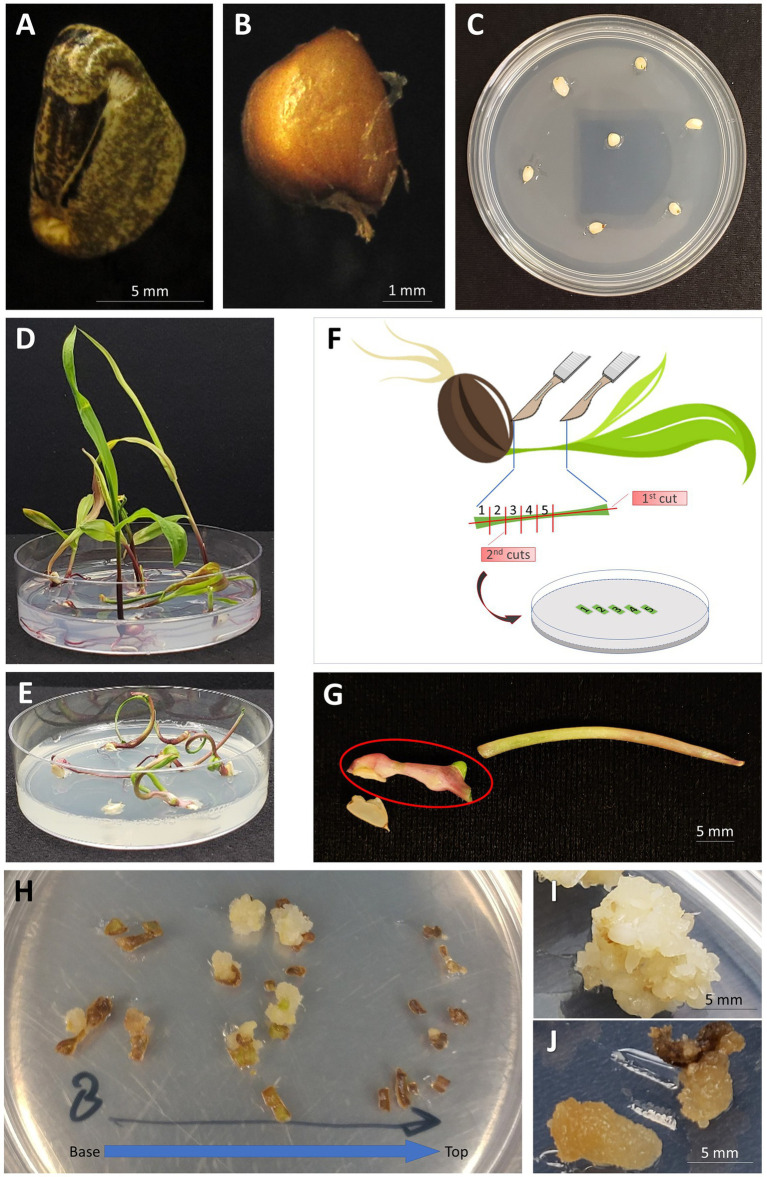
Generation of embryogenic callus from the leaf tissue of teosinte seedlings. **(A)** A teosinte seed with the hard casing intact; **(B)** A “naked” seed with the casing removed; **(C)** Naked teosinte seeds placed on growth medium for germination; **(D)** Germination of teosinte seeds on ½ MS growth medium (14days after sowing); **(E)** Germination of teosinte seeds on MSVS34-P2.2 growth medium (14days after sowing); **(F)** Illustration of cutting the leaf segment and placement on callus induction growth medium; **(G)** Whorl segment explant (indicated by red circle) collected from seedling germinated on MSVS34-P2.2 medium; **(H)** Callus forming from leaf pieces of plant grown on MSVS34-P2.2 (14days after callus induction). Leaf pieces are oriented from the base of the plant to the top; **(I)** Embryogenic callus capable of regeneration into a full plant (30days after callus induction); **(J)** Non-embryogenic callus that will not regenerate into teosinte plants (30days after callus induction).

Teosinte seeds (Figure 1A) were sterilized twice following the protocol described by Martinez and Wang (2009) before being placed on germination media (Martinez and Wang, 2009). About 10–50 seeds were submerged in 50ml of seed sterilization solution (Supplementary Table S1) in an Erlenmeyer flask with a stir bar. The flask was placed on a stir plate set to 220rpm. The sterilization of the seeds was carried out at room temperature (RT, 22–25°C) for 20min. The seeds were then rinsed three times with autoclaved deionized water (dH_2_O) to remove any residual bleach.

The sterilized seeds remained in sterile water for 5–18h at RT to soften its hard casing. Once softened, the casing was mechanically removed using a pair of clean nail clippers. The “naked” seeds (Figure 1B) were then sterilized using the same sterilization and rinse procedure as described above.

The “naked” teosinte seeds were germinated in a 25×100mm Petri dish containing germination media ½ MS or MSVS34-P2.2 (Supplementary Table S1) with seven seeds per plate (Figure 1C). The Petri dishes were left unwrapped and placed in a clean transparent plastic container [10cm (H)×30cm (L)×18cm (W)] with lid and incubated in a biological incubator at 28°C with 16/8h (day/night) photoperiod and 100–200μmol/m^2^/s light intensity measured using a LI-250A Light Meter (LI-COR Biosciences, Lincoln, Nebraska, United States). The teosinte plants were allowed to grow for 1–3weeks until they reached a height of ~5–9cm. If needed, the lids of Petri dishes were removed to allow plantlets to grow upright in the covered plastic container.

To prepare whorl segments (WSs) for callus culture, teosinte plantlets of appropriate sizes (Figures 1D,E) were harvested. Inside a laminar flow hood the germinated seedlings were cut to collect the WSs (Figure 1G) using a Number 10 Royal Tek surgical blade. The first cut was a lateral incision along the length of the plant creating two long segments (Figure 1F). Holding the two pieces together at the top of the plant using a pair of forceps, 1mm cuts were made perpendicular to the first cut. The 1mm pieces of leaf tissue were then evenly spaced onto callus induction media MSW57 or 605B (Supplementary Table S1, Figure 1F). The plates were left unwrapped, placed in a clean plastic box with lid as described above, and incubated at 28°C in the dark.

### Callus Induction, Maintenance, and Regeneration

Callus formation and subsequent growth occurred over the next 6–8weeks. WS pieces that began to form embryogenic callus were subcultured onto fresh callus induction media every 2–3weeks. Four to six weeks after callus induction, embryogenic teosinte calli were moved to maturation growth medium (13329iaa, Supplementary Table S1) containing BAP and indole-3-acetic acid (IAA) to promote shoot formation. Callus tissues on maturation media were cultured in the dark at 28°C. After 14days in the dark, the calli were moved into the light [16/8h (day/night), 100–200μmol/m^2^/s] at 28°C for 7–21days.

For rooting step, callus pieces with developing shoots were placed on rooting medium (272iba, Supplementary Table S1) with indole-3-butyric acid (IBA) to induce root growth. To prevent overgrowth, the calli were placed evenly on the rooting plates with no more than eight pieces per plate. The rooting process was carried out under light [16/8h (day/night), 100–200μmol/m^2^/s] at 28°C for 14–28days (subculture every 2weeks when needed). During the rooting step, callus with healthy and vigorously growing shoots were closely monitored. To encourage root formation, any excessive callus materials associated with these growing shoots were removed.

Once roots were established, healthy plants were transferred to soil. Excess growth medium associated with the roots was removed and roots were rinsed and cleaned with water. Plantlets were carefully separated using forceps and surgical blades. Rooted plants were transferred into 3 sq. in (19cm^2^) plastic insert (1801 deep inserts, T.O. Plastic, Clearwater, MN, United States) containing pre-wetted soil mix (Sungro^®^ Professional Growing Mix Sunshine^®^ Mix #1). The inserts were placed in a tray (27cm×54cm) and covered with a clear plastic humidity dome. The plants were grown in a growth chamber at 28°C with 12/12h (day/night) photoperiod and 250–320μmol/m^2^/s light intensity (~1.7m below the lights) for 7days. Developing plants were transplanted into one gallon (3.8L) plastic pots until maturation. Tassels began to form 8–12weeks after being placed in soil. To prevent cross-pollination, tassels were removed and stored separately in a beaker of water away from the silks of the teosinte. Plants were fertilized with Peters Excel 15-5-15 fertilizer as needed.

### Constructs for Biolistic Transformation

**Figure 2:**
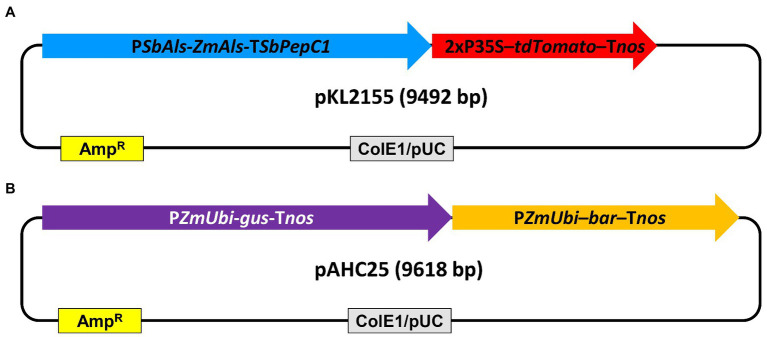
Schematic illustration of the plasmid DNA constructs used for teosinte transformation. **(A)** pKL2155, 9,492bp. P*SbAls*-*Als*-T*SbPepC1*, the sorghum acetolactate synthase gene promoter driving the mutant acetolactate synthase gene with sorghum *PepC1* gene terminator; 2xP35S-*tdTomato*-T*nos*, 2x CaMV 35S promoter driving *tdTomato* with *Agrobacterium* nopaline synthase gene (*nos*) terminator; **(B)** pAHC25 (Christensen and Quail, 1996), 9,618bp. P*ZmUbi*-*gus*-T*nos*, maize ubiquitin gene promoter (P*ZmUbi*) driving β-glucuronidase gene with the *nos* terminator; P*ZmUbi*-*bar*-T*nos*, P*ZmUbi* driving bialaphos resistance *bar* gene with the *nos* terminator; ColE1/pUC, high copy number origin of replication from *Escherichia coli*; Amp^R^, ampicillin resistance β-lactamase gene cassette.

Plasmid pKL2155 (Figure 2A; Supplementary Figure S1) and pAHC25 (Christensen and Quail, 1996; Figure 2B) were used for bombardment experiments. The plasmid pKL2155 (9,492bp) was designed in this work. The construct carries a mutant acetolactate synthase (*Als*) gene (Okuzaki et al., 2007) driven by sorghum *Als* gene (*SbAls*) promoter as selectable marker gene and a recombinant red fluorescent protein *tdTomato* gene (Shaner et al., 2004) driven by a double 35S promoter (Omirulleh et al., 1993) as a scorable marker gene (Figure 2A; Supplementary Figure S1). pKL2155 was constructed using Gibson assembly (Gibson et al., 2009). First, four different fragments were PCR amplified using primers, plasmid DNA templates (Supplementary Table S2a), and 2× Phusion High-fidelity DNA polymerase master mix (Thermo Fisher Scientific, MA, United States). For each fragment, 0.5μM primers and 5–10ng of plasmid DNA template were used for a 50μl reaction volume. Detailed thermocycling conditions are as follows: initial denaturation at 98°C for 30s, 35cycles of 10s at 98°C, 30s at 52.1–62.8°C, 30–75s at 72°C, and followed by final extension at 72°C for 5min. Annealing temperature and extension time varied depending on the fragment to be amplified (Supplementary Table S2a). PCR products were resolved on 1% agarose gel and corresponding DNA fragments were purified using the QIAquick PCR purification kit (Qiagen GmbH, Hilden, Germany) according to the manufacturer’s instruction. Amplified DNA fragments and the linear pJET1.2 DNA (Thermo Fisher Scientific, Waltham, MA, United States) were assembled using the HiFi DNA assembly master mix (New England Biolabs, Ipswich, MA, United States) according to the manufacturer’s protocol. Two microliters of the assembly mix were used for *Escherichia coli* (DH5α) transformation as described by Fronger and Hall (2007).

The plasmid pAHC25 is a 9,618bp in size and contains a selectable marker gene, *bar*, and a scorable marker gene, *gus*. Both genes are driven by separate maize ubiquitin promoters (Christensen and Quail, 1996).

Plasmid DNA was isolated and purified using QIAprep Spin Miniprep Kit (Qiagen GmbH, Hilden, Germany) per manufacture instruction.

### Preparation and Performing of Particle Bombardment

Two different methods were used for coating of gold particles with DNA for biolistic transformation. The spermidine/CaCl_2_ method was based on the procedure for biolistic transformation of maize immature embryo as described previously (Wang et al., 2020). Briefly, in an Eppendorf tube, 1.5mg surface-sterilized 0.6μm gold particles were mixed with 2–3μg of plasmid DNA, CaCl_2_ and spermidine. After coating, the gold/DNA complex was pelleted, washed by 100% ethanol (Decon 200 Proof Pure Ethanol Alcohol, Decon Labs Inc., King of Prussia, PA) and resuspended in 110μl of 100% ice-cold ethanol. For each shot, 10μl of gold/DNA suspension was aliquoted onto the center of each macrocarrier and allowed to air-dry prior to the bombardment. Typically, each bombarded plate received ~150μg of gold particles and 200–300ng of plasmid DNA in this work.

The majority of the bombardment experiments described in this work used a transfection reagent *Trans*IT^®^-2020 (Mirus Bio LLC, Madison, WI, United States) for coating DNA onto the gold particles following the protocol described in Miller et al. (2021) with modifications (Miller et al., 2021). Specifically, 1.5mg of surface-sterilized 0.6μm gold particles were homogenously suspended in 90μl of dH_2_O using an Ultrasonic Cleaner (Lab Safety Supply HB-3818B sonicator, Grainger, Lake Forest, IL, United States). Three μg of plasmid DNA (300ng/μl in dH_2_O) was added to the gold suspension and mixed by vortexing for 10s on high setting. Next, 1μl of *Trans*IT-2020 (ratio 0.33μl *Trans*IT-2020 per μg DNA) was added and the tube was vortexed on high for 15s. The Eppendorf tube was then vortexed in the upright position for 10min on low setting to allow contents to settle to the bottom of the tube. The Eppendorf tube was centrifuged at 9,391×*g* for 1min to pellet the gold. Using a pipette, the supernatant was removed without disrupting the gold/DNA pellet. Finally, 110μl of 100% ice-cold ethanol were added and the tube was then sonicated for 5s to ensure the homogenous suspension of gold/DNA. Ten microliter of gold/DNA were dispensed onto each macrocarrier and allowed to air-dry prior to the bombardment. Similar to spermidine/CaCl_2_ coating method, each bombarded plate using the *Trans*IT-2020 coating received ~150μg of gold particles and 300ng of plasmid DNA in this work.

For pre-bombardment osmotic treatment, approximately 1g of embryogenic callus derived from multiple leaf pieces of a single plant was placed onto the center of a MSW57 osmotic plate (Supplementary Table S1) in a circle ~2.5cm^2^ in diameter. The plates were incubated in the dark at 28°C for 4 to 24h prior to the bombardment. Bombardment of teosinte callus pieces was carried out directly on the MSW57 osmotic plates using the Biolistic PDS-1000/HE particle delivery system (Bio-Rad) inside a sterile flow hood as described (Wang et al., 2020). The specific parameters were 6cm target distance, 650psi rupture discs, and 26.5mmHg vacuum pressure.

### Selection and Regeneration of Transformed Callus

Bombarded calli were immediately moved from MSW57 osmotic medium onto fresh callus induction medium without any selective reagent (Supplementary Table S1) and incubated in the dark at 28°C. Seven to eleven days after the bombardment, the calli were moved onto callus induction medium with appropriate selection agent.

Teosinte callus bombarded with pKL2155 were cultured on callus induction medium containing 0.2mg/L of ethametsulfuron (EMS) after the resting stage. After the first round of selection, dead calli were discarded and the healthy ones were subcultured onto fresh selection media. Selection on callus induction media with 0.2mg/L EMS continued for 4–6weeks. Healthy EMS resistant calli were transferred onto maturation medium 11329iaa (Supplementary Table S1) containing the selective reagent 0.2mg/L EMS. Callus tissues on maturation media were cultured at 28°C in the dark. After 14days in the dark the maturation plates culturing EMS resistant calli were placed under the light [16/8h (day/night), 100–200μmole/m^2^/s] at 28°C for another 7–21days.

Teosinte tissues bombarded with pAHC25 were moved to MSW57 containing 2mg/L of bialaphos. Two weeks after the selection, healthy proliferating calli were moved onto callus induction medium containing 5mg/L of bialaphos. An optional third subculture to callus induction medium containing 5mg/L of bialaphos was used to increase the size of the transgenic calli if needed.

Developing shoots were placed on rooting medium 272iba (Supplementary Table S1) with either 0.2mg/L of EMS (for pKL2155) or 2mg/L bialaphos (for pAHC25) for root development. Rooted putative transgenic plants were acclimated and brought to maturity in a growth chamber as described in the section “Callus Induction, Maintenance and Regeneration” above.

### PCR Analysis

Genomic DNA was extracted from ~2cm^2^ clippings of teosinte leaf tissue using the protocol described in Edwards et al. (1991). PCR analysis of the genomic DNA was used to detect the presence or absence the transgenes in regenerated T0 teosinte plants and the progenies using the primers and conditions listed in Supplementary Tables S3a,b.

### Transgene Copy Number Estimation by Quantitative PCR Analysis

Genomic DNAs were extracted from teosinte leaf materials as described by Richards et al. (1994). A teosinte single copy gene, *ZvMEK1*, an ortholog of maize *ZmMEK1* (Hufford et al., 2021), was selected as a reference gene for the transgene copy number estimation. To generate standard curves, a 3,797bp plasmid (pKL2347) containing fragments of the reference gene *ZvMEK1* (261bp), *gus* gene (263bp) and *SbAls* promoter (299bp) was constructed using Gibson assembly (Gibson et al., 2009). These fragments were amplified by PCR using the primers listed in Supplementary Table S2b and the Q5 master mix (New England Biolabs, Ipswich, MA, United States). PCR products were purified using the QIAquick PCR purification kit (Qiagen GmbH, Hilden, Germany) and assembled with the linearized pJET1.2 DNA (Thermo Fisher Scientific, Waltham, MA, United States) using the HiFi DNA assembly master mix (New England Biolabs, Ipswich, MA, United States) according to the manufacturer’s instruction.

Transgene quantification was carried out using TaqMan PCR. Quantitative PCR (qPCR) primers and probes were designed using the PrimerQuest™ Tool (IDT, Coralville, IA, United States), and the probes with compatible dyes (HEX for *ZvMEK1*; FAM for *gus* and *SbAls* promoter) and double quenchers (ZEN/Iowa Black™ FQ) were synthesized by IDT (Coralville, IA, United States). Each qPCR was performed in a 20μl reaction volume with the following ingredients (final concentration): 0.5μM *ZvMEK1* primer pair, 0.5μM *gus* or *SbAls* promoter primer pairs, 0.25μM probe for *ZvMEK1*, 0.25μM probe for *gus* or *SbAls* promoter, 0.01pg–1ng pKL2347 plasmid DNA (2.57×10^3^–2.57×10^8^ copies) or 10–20ng of teosinte genomic DNA, and 1×PrimeTime™ Gene Expression Master Mix (IDT, Coralville, IA, United States). Real-time PCR was carried in an Mx3005p qPCR system (Agilent, Santa Clara, CA) using the fast-cycling condition as instructed by the manufacturer: 3min at 95°C for polymerase activation followed by 40cycles of amplification with 5s at 95°C for denaturation and 30s at 60°C for annealing/extension. All reactions were performed with four technical replicates. Transgene copy numbers were estimated using the standard curves generated by the pKL2347 DNA copy numbers and the qPCR cycle threshold values (*C*_t_). The amplification efficiencies were 98% for the *SbAls* primers (*r*^2^=0.999), 107% for the *gus* primers (*r*^2^=0.999), and 98–107% for the *ZvMEK1* primers (*r*^2^=0.999). Because the single copy reference gene has two alleles in the genome, a single copy transgene would have a 1:2 ratio of *gus* or *SbAls* promoter to *ZvMEK1*.

### Southern Blot Hybridization Analysis

Genomic DNA was extracted from leaf tissue of T0 and T1 plants using cetyltrimethylammonium bromide (CTAB) method (Murray and Thompson, 1980). Fifteen micrograms of gDNAs were digested with SacI enzyme and separated on a 0.9% (w/v) agarose gel by electrophoresis. The DNAs were transferred to and cross-linked to a membrane (BrightStar Plus; Thermo Fisher Scientific, Waltham, MA, United States). The membrane was prehybridized using Church’s buffer (Church and Gilbert, 1984) for 4h at 65°C. The probe was designed to detect the *gus* gene. It was amplified by PCR from the plasmid pACH25 using primers Gus-F (TTG GGC AGG CCA GCG TAT CGT) and Gus-R (ATC ACG CAG TTC AAC GCT GAC). The 421bp product was purified and subsequently labeled with ^32^P-dCTP using the Prime-it II kit (Agilent Technologies, Santa Clara, CA, United States). The labeled probe was purified (Illustra Probequant columns, VWR, Radnor, PA, United States) and incubated with the DNA membrane overnight at 65°C. The membrane washing and film development were done following standard protocols (Green and Sambrook, 2012).

### Seed Germination for Progeny Analysis

Teosinte seeds do not readily germinate after harvest (Mondrus-Engle, 1981). To germinate naturally aged seeds (minimum 3months after harvest in this work) for progeny analysis, seeds were sterilized and the hard casing were removed as described above in “Seed sterilization and explant preparation.” A sterile 100mm×25mm Petri dish was lined with one piece of 7cm or 12cm diameter Whatman filter paper in the flow hood. Autoclaved distilled water was used to wet the filter paper, with a 1ml pipette. The sterile “naked” seeds were placed on the pre-wetted filter paper. The petri dish was wrapped with a porous tape and incubated at 26°C in the dark for 4–7days until sprouting. The germination is monitored daily. To prevent drying of the germinating seedlings, additional sterile water was added, as needed, to the filter paper to keep it moistened constantly. The sprouts with established roots and shoots were planted into soil as described above in “Callus induction, maintenance, and regeneration.”

To accelerate germination of freshly harvested and delicate teosinte seeds produced from tissue culture plants grown in a confined growth chamber, germination was carried out *in vitro* without surface sterilization using bleach or ethanol. The seeds were soaked in a filter-sterilized gibberellic acid (GA3) solution (1mg/L), at 26°C for 24h on the lab bench under ambient lighting. The hard casing of seed was aseptically removed. The “naked” seeds were placed, embryo axis up, in a ½ MS germination medium (Supplementary Table S1) supplemented with 1mg/L GA3. The plates were incubated in a biological incubator at 28°C with 16/8h (day/night) photoperiod and 100–200μmol/m^2^/s light intensity. The germination could be observed as early as 10days after the GA3 treatment.

### Phenotyping

Callus tissue and the T1 seeds of pKL2155 were screened for *tdTomato* gene expression using fluorescent microscope (Olympus SZH10 stereo microscope with Texas red filter, EX 535–585nm, EM 605–690nm) or using a NIGHTSEA dual fluorescent protein flashlight and filter glasses (NIGHTSEA LLC, Lexington, MA, United States).

Putative transgenic T0, T1, and T2 plants generated from the bombardment of pAHC25 were phenotyped using GUS stain as described (Jefferson et al., 1987).

pAHC25 transgenic T2 progeny was tested for herbicide resistance using the protocol described previously (Frame et al., 2006). For screening of glufosinate tolerance, fully expanded leaves from 2-week-old teosinte seedlings were used. Glufosinate solution (150mg/L glufosinate plus 0.1% Tween 20; freshly prepared from the herbicide Liberty®, BASF, Ludwigshafer, Germany) was applied onto one-third of the leaf surface (from tip of the leaf). Q-tips soaked with the glufosinate solution were used to gently rub both sides of the leaves to ensure the herbicide contact and penetration. Plants were assessed for damage 2days after the leaf painting.

### Statistical Analysis

Statistical analysis for plant regeneration using different germination and callus induction media was conducted using a *z*-score test for two independent proportions.^3^ Pairwise comparisons were made for all six combinations among the four different treatments.

## Results

### Establishing Embryogenic Callus Culture From *in vitro* Grown Seedlings

An efficient regeneration protocol is a pre-requisite for establishing a genetic transformation protocol using a tissue culture system. To overcome the limited production of immature embryos in teosinte plants, we used seedlings germinated from mature seeds as an alternative starting material. Unlike maize, teosinte is resistant to inbreeding. As a result, the seed accessions exist as populations (Fukunaga et al., 2005). Therefore, it is expected that the seed germination and callus initiation rates may differ between plants. In this study, we chose to use seed accessions Ames 21785 and Ames 21789 acquired from USDA Agricultural Research Service seed repository and *Zea parviglumis* seeds from Restoration Seeds (Talent, OR, United States). We chose the Restoration Seeds (RS) for its bulk quantity needed in the experiments.

To ensure uniform seed germination and to minimize contamination *in vitro*, the hard casing covering teosinte seed was removed. Twice disinfected (before and after removal of the casing) “naked” seeds were then placed on germination media (Figures 1A–C). Two types of germination media were evaluated (Supplementary Table S1). The ½ MS medium was a simple MS/sucrose-based medium, and MSVS34-P2.2 was MS-based medium supplemented with maltose and growth hormones cytokinin (BAP) and auxin (picloram). MSVS34-P2.2 was modified based on MSVS34 medium used by Sidorov et al. (2006) for maize seedling-derived callus (Sidorov et al., 2006).

The “naked” teosinte seeds could be germinated from both media. Table 1 shows typical germination rates for RS seeds, with 75.7% on ½ MS and 65.7% on MSVS34-P2.2. Because of the presence of picloram, plants germinated from the MSVS34-P2.2 medium grew abnormally with curled and stunted shoots (Figure 1E) when compared with the plants from the ½ MS medium (Figure 1D).

**Table 1:** Summary of plant regeneration using different germination and callus induction media.

Trtmnt	Germination				Callus induction				Regeneration^*^	
	Germination media	No. of seeds sterilized	No. of seeds germinated	Germination rate (%)	Callus induction media	Total number of WS	No. of WS forming callus	Callus induction rate (%)	No. of WS regenerated	Plant regeneration rate (%)
1	1/2 MS	37	28	75.68	MSW57	11	11	100.00	0	0.0^a^
2					605B	12	11	91.67	2	18.2^ab^
3	MSVS34	35	23	65.71	MSW57	11	10	90.91	5	50.0^bc^
4					605B	11	10	90.91	7	70.0^c^

* *Regeneration rates with same letters are not statistically significantly different*.

*WS, whorl segment*.

Teosinte seedlings were first dissected to 2–3cm-long whorl segments (Figures 1F,G). Each WS was considered as an explant. Then the segments were cut into 1mm pieces and placed on callus induction media (Supplementary Table S1). To test and track the degrees of callus initiation ability, these pieces were arranged on the media according to their anatomical positions on the segment (Figures 1F,H). Two to three weeks after the initiation, calli were readily formed from pieces that contain shoot apical meristematic region of the first node of the teosinte seedling (Figure 1H and Supplementary Figure S2A). While not all pieces derived from the same plants formed callus, most of RS seedlings tested on both media produced various callus cultures as embryogenic callus (Figure 1I, Supplementary Figure S2B) and non-embryogenic callus (Figure 1J, Supplementary Figure S2B). The callus induction frequency was calculated as percentage of callus-producing WSs in total dissected WSs used in the experiment (Table 1).

### Optimizing Callus Regeneration Frequency

**Figure 3:**
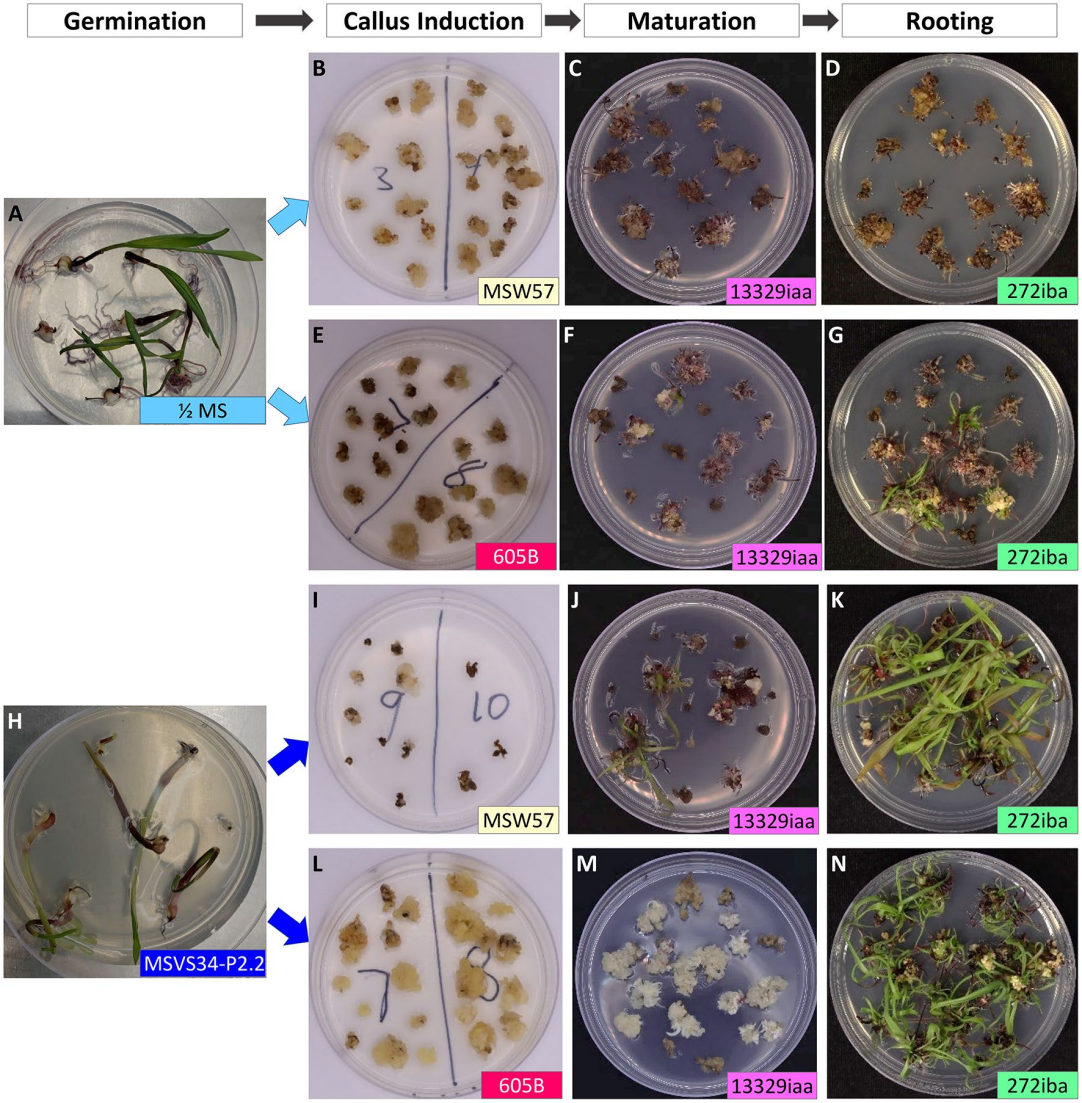
Effects of germination and callus induction media on teosinte callus induction and regeneration. Disinfected, “naked” teosinte seeds germinated on ½ MS medium **(A)** or MSVS34-P2.2 medium **(H)**; **(B–D)** leaf pieces from ½ MS germinated seedlings were cultured on callus induction medium (MSW57), maturation medium (13329iaa), and rooting medium (272iba); **(E–G)** leaf pieces from ½ MS germinated seedlings were cultured on callus induction medium (605B), maturation medium (13329iaa), and rooting medium (272iba); **(I–K)** leaf pieces from MSVS34-P2.2 germinated seedlings were cultured on callus induction medium (MSW57), maturation medium (13329iaa), and rooting medium (272iba); **(L–N)** leaf pieces from MSVS34-P2.2 germinated seedlings were cultured on callus induction medium (605B), maturation medium (13329iaa), and rooting medium (272iba). Approximate ages of the plant materials were: under the labels of Germination **(A,H)**, 21days after sowing; Callus Induction **(B,E,I,L)**, 43days after callus induction; Maturation **(C,F,J,M)**, 21days after placing on Maturation medium; and Rooting **(D,G,K,N)**, 12days after placing on rooting medium.

To improve regeneration ability of callus culture, we compared two different callus induction media MSW57 (Sidorov et al., 2006) and 605B (modified from Masters et al., 2020). Both were MS/sucrose-based media supplemented with silver nitrate and auxin (picloram in MSW57 and dicamba in 605B, Supplementary Table S1). Leaf nodal pieces from seedlings germinated in ½ MS or MSVS34-P2.2 were divided equally and placed on either MSW57 or 605B for callus induction (Figure 3). As shown in Table 1, greater than 90% of callus induction could be achieved for all four treatments. The callus generated on each growth medium was morphologically consistent, displaying a range of phenotypes including type I (Figure 1I, Supplementary Figure S2B), pre-type II embryogenic callus and watery, loose-structured non-embryogenic callus (Figure 1J, Supplementary Figure S2B). Both MSW57 and 605B callus induction media performed well regardless of germination media, generating callus in >90% of the WSs placed on each medium. However, the regeneration rates were different between seedlings germinated on MSVS34-P2.2 (50 and 70%) vs. ½ MS (0 and 18%). Statistical analysis using two-tailed *z*-test of difference in proportion indicated that regeneration rates of treatments 3 and 4 were significantly higher than that of treatment 1 (*p*<0.01, Table 1 and Supplementary Table S4). This suggests that using MSVS34-P2.2 as a germination medium can result in higher plant regeneration rates with either MSW57 (treatment 3) or 605B (treatment 4) as callus induction media (Table 1 and Supplementary Table S4). The regeneration rates of treatments 2 and 4 were significantly different at the level of *p*<0.05, but not at *p*<0.01. The regeneration rate differences between treatments 2 and 3 as well as treatments 3 and 4 were not significant (Supplementary Table S4). Here, regeneration rate was measured as the number of rooted shoots divided by the total number of WSs used for callus initiation.

Plants regenerated from the experiments were moved to soil and brought to maturity. Fertile seeds were successfully recovered from regenerated plants. A portion of the regenerated teosinte plants displayed a tassel-ear phenotype, but seeds could be recovered from these plants. These results showed that the inclusion of auxin in germination medium plays critical role in plant regeneration in teosinte.

The embryogenic callus (Figure 1I) forming from WS pieces was often lighter in color and more dense than non-embryogenic callus (Figure 1J), which was often watery and darker. Callus cultures of similar appearance and derived from the same plant did not always maintain the same ability to regenerate into fertile plants. Supplementary Figure S3A shows regeneration ability from two callus lines that were derived from two pieces originated from one single WS. Initially, both pieces produced vigorously growing calli consisting of both embryogenic and non-embryogenic types. Interestingly, these two morphologically similar callus lines displayed markedly different regeneration ability.

Teosinte callus could retain its regeneration competency for several months, but the rates diminished with age of the cultures. For example, in one experiment we observed that callus of Ames 21785 retained 100% regenerability after 8months but less than 50% of the callus could be regenerated after 15months (Supplementary Figure S3B).

Our methods included a 3-week treatment on maturation medium (13329iaa, Supplementary Table S1) when the callus cultures were transitioned from dark to light for shoot induction. The inclusion of this step was essential to ensure the formation of shoots, whereas in the absence of this step no plants were recovered from the callus culture (Supplementary Figure S3B).

### Biolistic-Mediated Teosinte Transformation

Before starting our transformation experiments, we conducted experiments to create kill curves using the selective reagents on non-transformed callus tissues. We tested two selective reagents (bialaphos for the *bar* gene, and EMS for the *Als* gene) that are commonly used in maize transformation and determined the efficacy of each reagent in teosinte. Our results showed that 5mg/L of bialaphos and 0.2mg/L of EMS were sufficient to arrest callus growth in teosinte (Supplementary Figure S4).

Two plasmid DNA constructs, pKL2155 (Figure 2A, Supplementary Figure S1) or pAHC25 (Christensen and Quail, 1996; Figure 2B) were used for bombarding teosinte embryogenic callus cultures. Callus chosen for the bombardment experiments were healthy and proliferate actively with some embryogenic sectors (Figure 1I). The callus cultures were treated in osmotic containing medium prior to the bombardment (Supplementary Table S1; Wang et al., 2020). Majority of the experiments used a transfection reagent *Trans*IT®-2020 for DNA/gold particle coating (Miller et al., 2021). Transient transformation comparison has shown that DNA/gold microprojectiles coated using *Trans*IT®-2020 performed equally well compared to that coated using the traditional spermidine/CaCl_2_ reagents (Supplementary Figure S5). Because *Trans*IT®-2020 coated DNA/gold suspension formed less aggregates, it was easier to handle during the microprojectile loading process.

**Figure 4:**
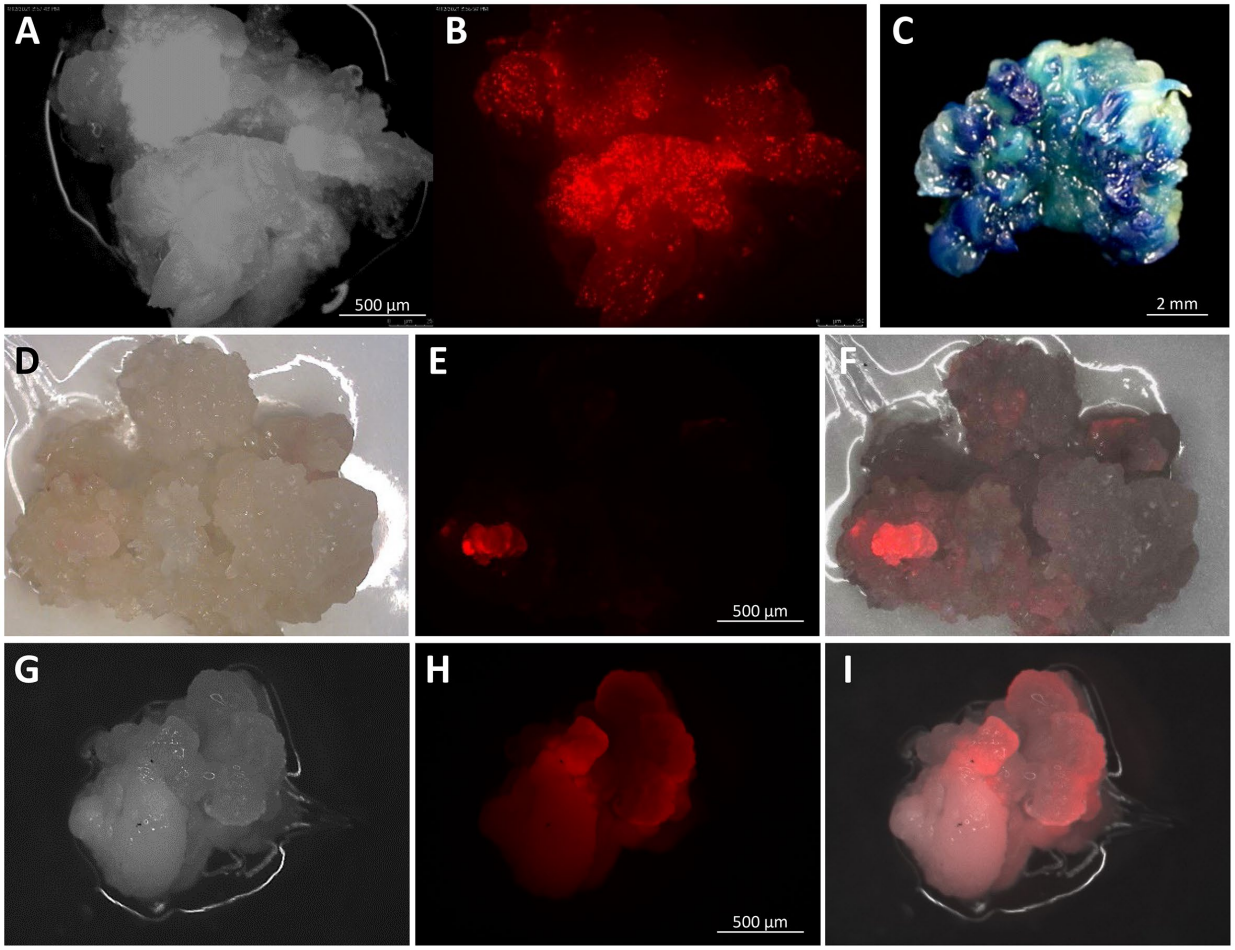
Embryogenic callus at different stages after particle gun bombardment. Embryogenic callus at 4days after bombardment with pKL2155 under a bright field **(A)** and under red fluorescent filter **(B)** showing transient expression of the *tdTomato* gene; a stable pAHC25 callus event expressing the *gus* gene **(C)**; a pKL2155 callus event expressing the *tdTomato* gene, 63days **(D–F)** and 154days **(G–I)** post bombardment under bright field **(D,G)**, red fluorescent filter **(E,H)**, and merged **(F,I)**. Images were taken using Olympus SZH10 stereo microscope with Texas red filter (EX 535–585nm, EM 605–690nm).

Table 2 summarizes 10 independent bombardment experiments carried out within a 5-month period. All the experiments were performed using embryogenic callus culture generated from teosinte RS seedlings. Each experiment used bulked callus pieces derived from several WSs (ranged from 1 WS in Exp #3 and #7, and 15 WSs in Exp #6). The age of callus cultures ranged from 34days (Exp #6) to 104days (Exp #1). The bombardment used pKL2155 plasmid, which contains the *tdTomato* gene for visual screening and the herbicide-resistant *Als* gene for transgenic plant selection. Transient *tdTomato* expression could be visualized 4days after the bombardment on most callus tissues (Figures 4A,B). Four to six weeks after incubating the callus on selection (callus induction media containing 0.2mg/L EMS), proliferating calli could be identified. Some resistant callus pieces displayed strong red fluorescence (Figures 4D–I). The expression of the *tdTomato* gene on callus tissue was strong; the red fluorescence could be visualized readily by using a hand-held NIGHTSEA BlueStar flashlight and filter glasses (NIGHTSEA LLC, Lexington, MA, United States).

**Table 2:** Summary of 10 biolistic transformation experiments using RS callus culture and pRK2155.

EXP ID	Date of seed germination	Date of bombardment	Age of culture (days)	No. of WS pooled & bombarded	No. of transgenic events	Transformation frequency (%)
1	2020.10.21	2021.2.2	104	4^*^	2	50.00
2	2020.12.8	2021.2.24	78	3	0	0.00
3	2020.12.28	2021.2.24	59	1	0	0.00
4	2021.1.7	2021.2.24	49	12	1	8.33
5	2021.1.16	2021.2.24	40	11	1	9.09
6	2021.2.25	2021.3.30	34	15	1	6.67
7	2021.2.26	2021.3.30	33	1	0	0.00
8	2021.3.3	2021.3.30	27	10	0	0.00
9	2021.3.10	2021.4.8	29	8	0	0.00
10	2021.3.4	2021.4.14	41	11	0	0.00
Total^#^				72	3	4.17

* *Each plate was bombarded twice in this experiment*;

# *Total numbers include data from Exp 2 to Exp 10*;

*RS, Restoration Seed; WS, whorl segment*.

**Figure 5:**
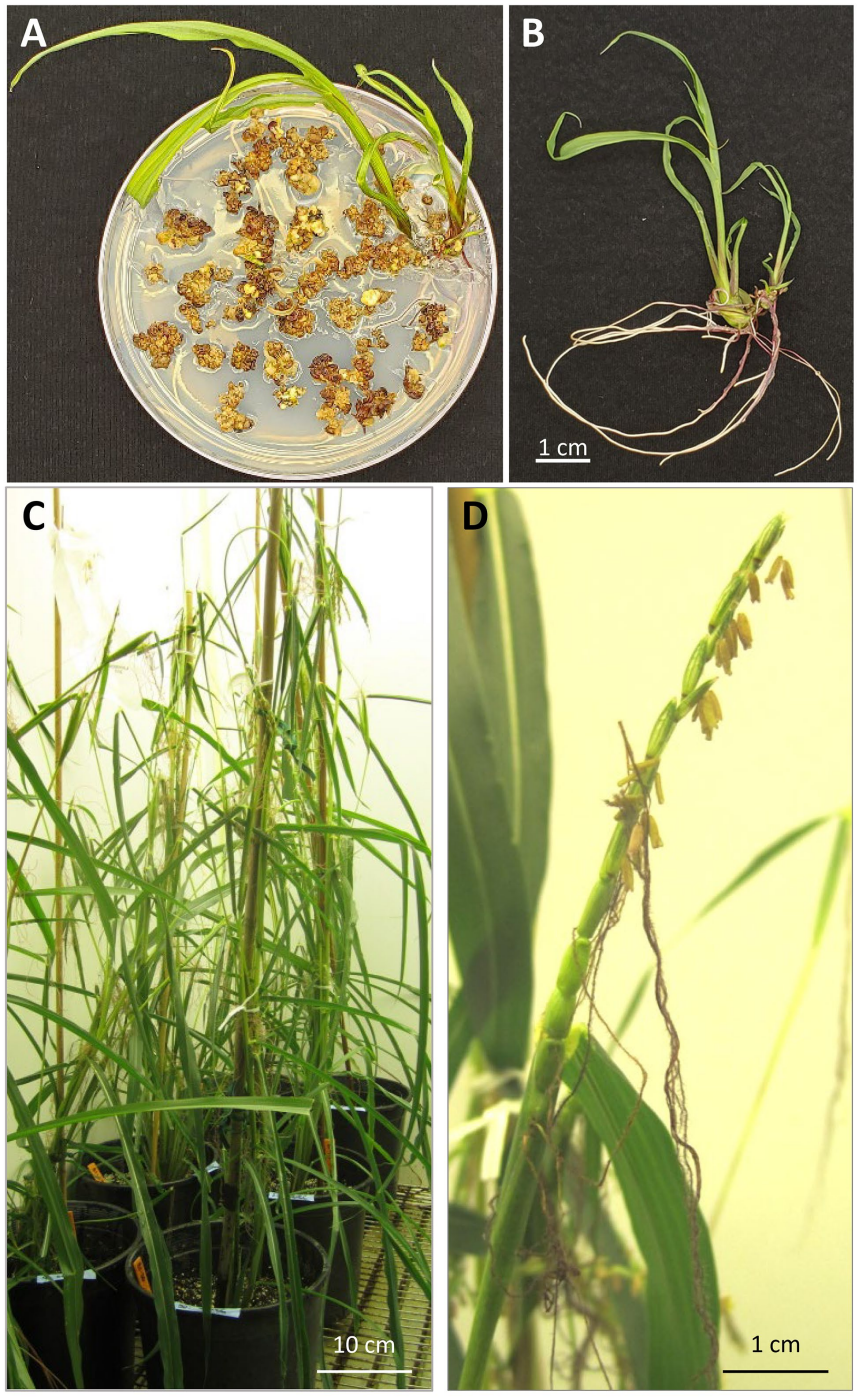
Regeneration and growing of T0 plants. **(A)** Regenerating plants on a rooting medium containing selective agent 0.2mg/L of ethametsulfuron; **(B)** teosinte regenerated from embryogenic callus displays an independent shoot and root system; **(C)** regenerated T0 plants in a growth chamber; **(D)** “tassel-ear” on regenerated teosinte plant that has both male (stamens) and female (gynoecia) at the top of the plant.

**Figure 6:**
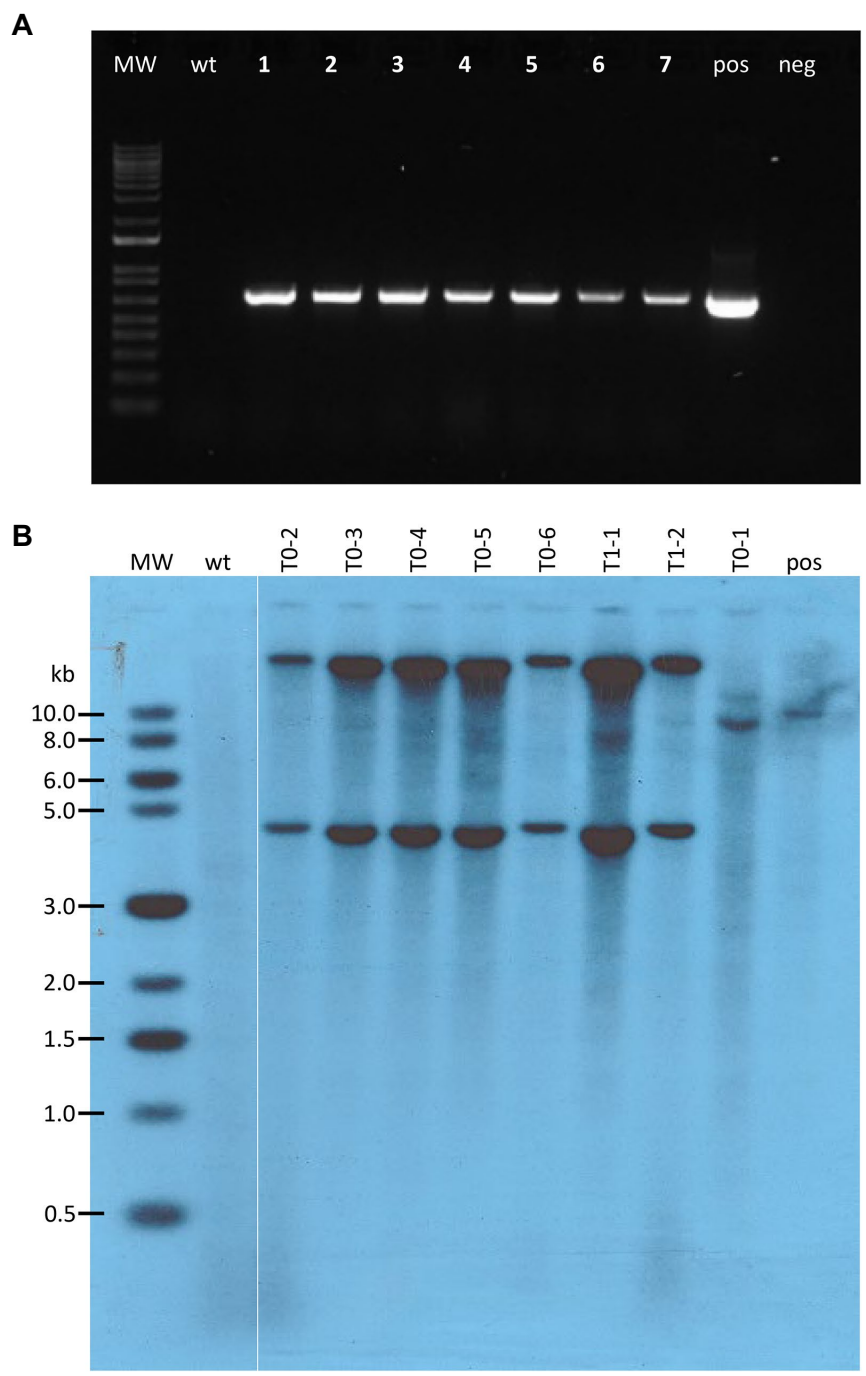
Molecular analysis of transgenic teosinte plants. **(A)** PCR results from genomic DNA extracted from pKL2155 transgenic T0 leaf tissue. Bands of 696bp indicate the *tdTomato* red fluorescent marker gene is present in the genomic DNA samples; MW, 1 Kb Plus DNA ladder; wt, wild-type non-transgenic teosinte; lanes 1–7, seven genomic DNA samples representing five independent pKL2155 events; pos, pKL2155 plasmid; neg, no DNA negative control; **(B)** Southern blot analysis of six T0 and two T1 transgenic plants derived from one pAHC25 event. Fifteen micrograms of genomic DNA were digested with *Sac*I restriction enzyme, which cuts only once in the plasmid pAHC25. The membrane was hybridized with a 421bp, ^32^P-labeld *gus* probe. MW, 2-Log DNA Ladder; wt, wild-type teosinte; pos, wild-type teosinte genomic DNA spiked with pACH25 plasmid.

A total of five EMS resistant callus pieces were generated and regenerated (Table 2). They were derived from four independent bombardment experiments. A total of 49 rooted plants were produced from these five events (Figures 5A,B and Supplementary Table S3c). PCR analyses were performed on 27 T0 plants that survived in soil using three different pairs of primers (Supplementary Tables S3a,b). All 27 plants were tested positive for at least one transgene from pKL2155 (Figure 6A, Supplementary Figure S6 and Supplementary Table S3c). Transformation frequency (TF) was calculated as the percentage of PCR confirmed T0 event of the total callus-producing WSs bombarded. TF of the four successful bombardment experiments ranged from 6.67% (Exp #6) to 50% (Exp #1). Note that in Exp #1, each plate was bombarded twice using the same parameters. Excluding Exp #1, average TF from nine independent experiments was 4.17%.

Quantitative PCR analysis was used to estimate the transgene copy numbers in the five T0 events. We selected a single copy *ZvMEK1* gene, an ortholog of maize *ZmMEK1* (Hufford et al., 2021), as the reference gene for the qPCR analysis. The qPCR analysis for pKL2155 events examined the transgene region containing the *SbAls* promoter. We chose to analyze the *SbAls* promoter region to avoid detecting endogenous *Als* gene in teosinte. Estimated transgene copy numbers in the five pKL2155 T0 events were low (Table 3). Two events (2155-B and 2155-E) have approximately one copy *Als* transgene; one event (2155-A) has two copies; two remaining events (2155-C and 2155-D) have less than one copy. The less than one copy number could be attributed to the possible mosaic nature of the transgene in these T0 plants.

**Table 3:** Transgene copy number analysis.^*^

Event ID	Generation	# Plants analyzed	Copy number
			Mean±SD
2155-A	T0	2	1.61±0.28
2155-B	T0	2	0.73±0.58
2155-C	T0	2	0.46±0.06
2155-D	T0	1	0.29
2155-E	T0	3	0.99±0.52
2155-A-1	T1	4	1.49±0.73
pAHC25-1-1-1	T2	2	3.81±0.40

* *A 299bp sequence in SbAls promoter region was used for 2155 events; a 263bp sequence in gus gene coding region was used for pAHC25 event. See section Materials and Methods and Supplementary Table S2b for details*.

**Figure 7:**
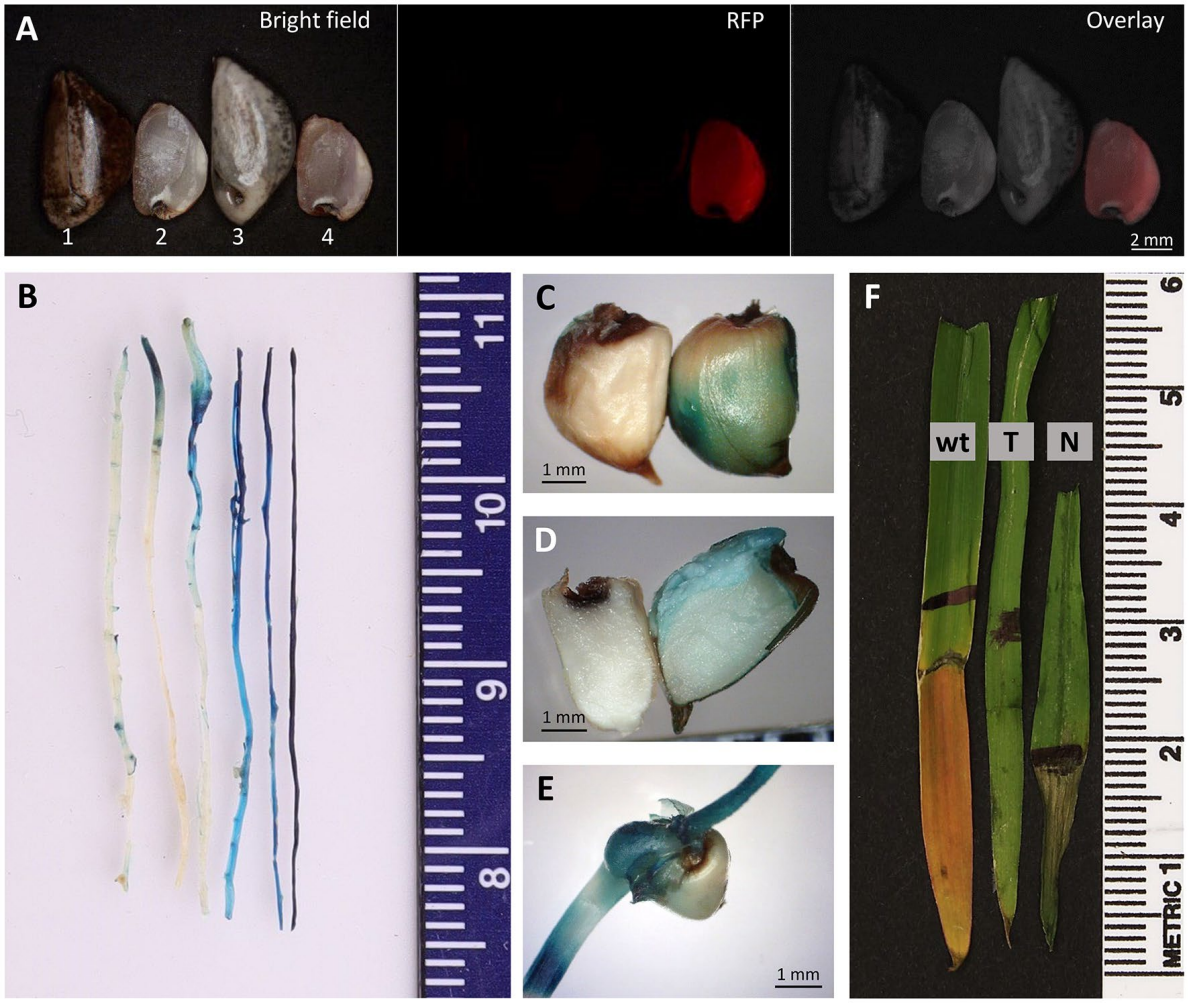
Progeny analysis. **(A)** Images of intact (#1 and #3) and “naked” (#2 and #4) teosinte seeds of Bright field (left), RFP (center) and Overlay (right). “Naked” transgenic T1 seed of pKL2155 (#4) appears pink in Bright field. Seeds #1 and #2, wild-type teosinte; Seeds #3 and #4, transgenic event 2155-A T1 seeds. GUS assay performed on roots **(B)**, intact “naked” seeds **(C)**, half “naked” seed **(D)** and germinating shoot **(E)** of T2 transgenic teosinte of pAHC25 event. Blue color indicates the *gus* gene expression. **(F)** Herbicide leaf painting assay. Portion of the leaf surface was applied with 500mg/L glufosinate plus 0.1% Tween-20 using a Q-tip. Image was taken 2days after the herbicide-application. wt, wild-type teosinte seedling; T, herbicide resistant T2 transgenic seedling; N, herbicide sensitive T2 null segregant.

qPCR analysis was also performed on four seedlings germinated from the 2,155-A event (Table 3), showing a similar copy number (1.49±0.73) in the T1 plants compared to its T0 parent (2155-A, 1.61±0.28). Transgene *tdTomato* expression in the progeny was monitored in the T1 seeds. Red fluorescence could be readily detected and visualized under a fluorescent microscope on the “naked” seed but not on the intact seed (Figure 7A).

Bombardment experiments using pAHC25 were carried out using callus derived from Ames 21789 seedlings. Out of three bombardments, one GUS-positive event (Figure 4C) was produced. This event was self- or sib- pollinated in a growth chamber (Figures 5C,D). Southern blot hybridization analysis was performed on six T0 plants and two T1 plants (Figure 6B). Five of the six T0 plants showed two dominant bands hybridized with the ^32^P-labled *gus* probe, suggesting at least two transgene insertions in the genome (Figure 6B). The different patterned T0-1 plant did not survive the soil transfer; therefore, no further investigation was conducted.

qPCR analysis of the *gus* transgene was performed on four T2 plants of the pAHC25-1 event: two GUS-positive and two GUS-negative plants. It revealed that the two GUS-negative plants indeed did not have the transgene, whereas the two GUS-positive plants have ~4 transgene copies integrated in the teosinte genome (Table 3). This discrepancy likely reflects the zygosity of T0 vs. T2 transgenic plants. If the T2 plant was a homozygous for the transgene, its copy number is likely doubled compared to the T0 plant, which was a hemizygous for the transgene.

Phenotyping of the pAHC25-1 T1 progeny showed that 27 (77.1%) were GUS-positive and eight (22.9%) were GUS-negative. T2 progeny analysis of 19 seedlings showed 14 (73.7%) GUS-positive and five (26.3%) GUS-negative plants (Figures 7B–E). All GUS-positive plants showed resistant to herbicide glufosinate while the GUS-negative plants showed necrosis on the portion of the leaf surface where the herbicide was applied (Figure 7F).

**Figure 8:**
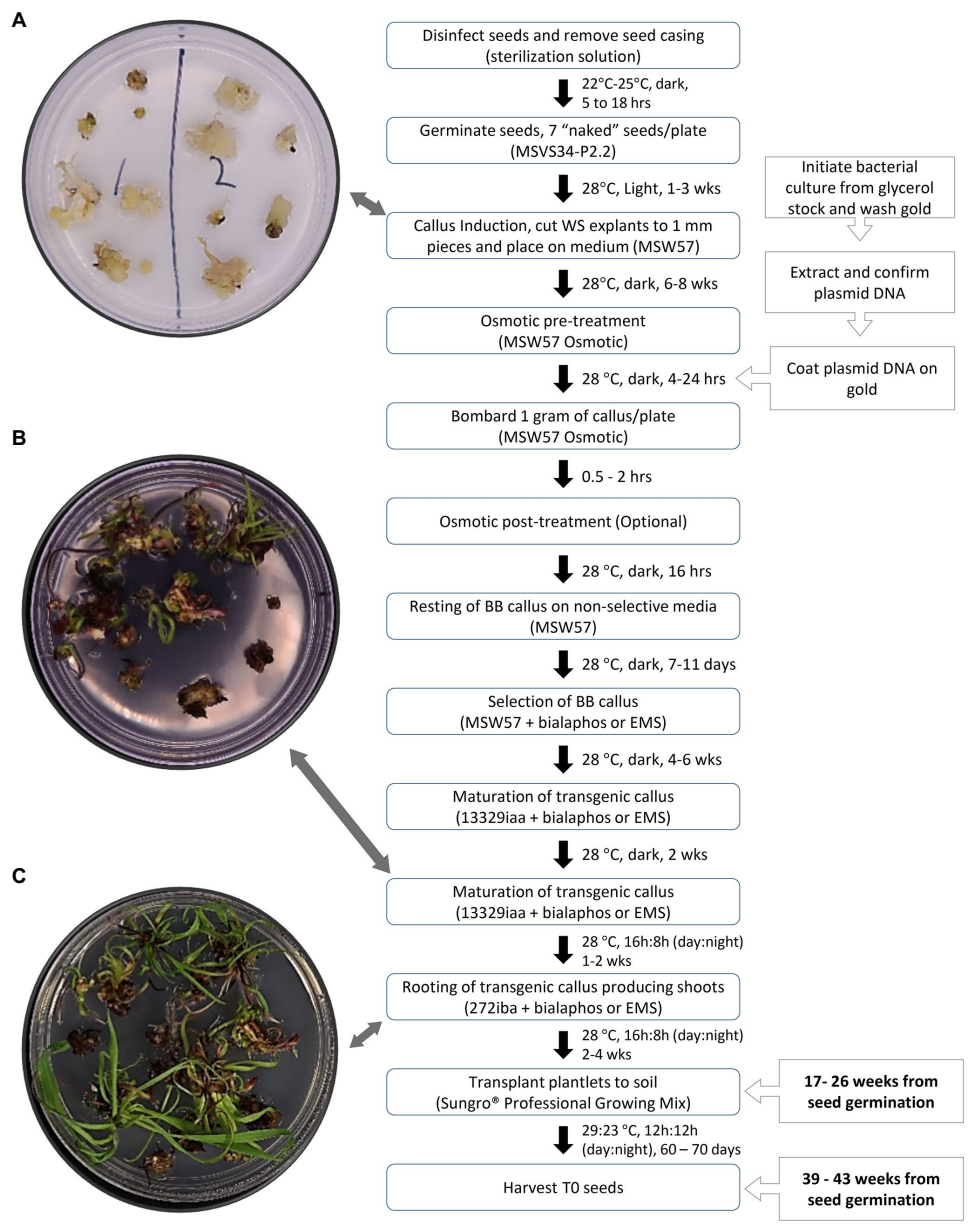
Timeline and flowchart of biolistic-mediated DNA delivery in teosinte (*Zea mays* ssp. *parviglumis*). **(A)** Callus initiated from leaf pieces; **(B)** Shoots at the end of maturation; **(C)** Root forming plantlets. WS, whorl segment; EMS, ethametsulfuron.

Figure 8 presents a flowchart of our current transformation process used to produce embryogenic callus and transgenic T0 events. The process involves typical steps required for most biolistic-mediated transformation. Bombardment experiments can be conducted 6–8weeks after the initiation of the callus from whorl segments. Four to six weeks after the selection, herbicide-resistant embryogenic callus culture can be placed onto maturation media with selection agent for shoot induction. Once the cultures are moved to light, rooted plantlets can be produced within 3–4weeks. The entire process takes approximately 4–7months for obtaining transgenic plantlets from the day of seed germination.

## Discussion

This work represents the first documentation of the successful regeneration of teosinte (*Zea parviglumis*) from mature seed-derived shoot segments, and the production of fertile transgenic plants using the biolistic method. The total duration from seed to transgenic seed takes approximately 8–10months, similar to a typical maize transformation protocol using immature embryos (Wang et al., 2020). Ten independent bombardment experiments are reported in this work. Six experiments generated no transgenic plants. Out of four successful experiments, three had similar transformation frequency (TF), that is, 6.67, 8.33, and 9.09% (Table 2). In one of the four experiments (Exp #1), each plate was bombarded twice. This experiment gave a TF of 50%. It has been reported previously in other plants that more DNA delivery *via* bombardment could yield higher transformation frequencies (Lowe et al., 2009; Jackson et al., 2012). However, there were not enough experiments performed in this work to conclude that this high TF was due to more DNA delivery in plant cells.

Unlike maize, teosinte exists in populations; hence, plants in each accession perform differently under same conditions. We observed large variations in teosinte tissue culture responses and transformation competency from different plants. The large variation in TFs reported here could partially be due to the heterogenous nature of the seeds used in each experiment; some had higher regeneration ability and transformation competency, the ability to receive DNA and integrate transgene into the genome, than others.

Molecular analyses, including PCR and qPCR, were performed on five T0 events and one T1 event of pKL2155 transformants. Bringing large number of teosinte plants to maturity can be challenging when plant growth facility spaces are limited. When growing multiple transgenic maize plants of similar age in one confined growth room, one can cover the female flower and remove the male flower to minimize pollen cross-contamination occurring between transgenic plants. However, this practice does not apply for growing teosinte plants. Due to the nature of the plant, the best practice for producing teosinte seeds is to put a few clonal plants together in one small growth chamber and let the plants to cross-pollinate each other. Transgenic plants from different events cannot grow together to maturity in a confined room, because of high likelihood of cross-contamination between plants. In this work, we performed transgenic analysis in all T0 plants. We were able to perform genotyping and phenotyping on T1 and T2 progeny of one pAHC25 transgenic event, demonstrating the transmission of transgenes into its progeny. We were also able to perform analysis on T1 plants from one pKL2155 event (2155-A) that were freshly obtained from a recent germination experiment, showing the similar transgene copy numbers as its parent. The T1 seed of the event 2,155-A clearly showed the inheritance and expression of the transgene *tdTomato*. Based on this observation, and experiences from producing other transgenic maize and rice plants in our lab, we are confident that the other four pKL2155 events can pass the transgenes into their progenies.

Because of the non-uniform nature of the teosinte seeds, it is important to consider working with multiple embryogenic callus lines (callus derived from several different plants) to achieve genetic transformation. During early stage of this work, we often focused on one or two individual embryogenic callus lines that were bulked up for evaluating various bombardment parameters and DNA/gold coating procedures. While we were able to observe many transient events after bombardment and gained valuable insights using these unique “bulked callus” stock, we were unable to recover any stable transgenic events. Because we do not know what factors contribute to transformation competency, we prefer to collect callus materials generated from multiple plants for bombardment experiments. Compared to bulking up one single callus line, the collection of multiple callus line materials would allow us to conduct bombardment experiments on newly established callus lines sooner, within a few weeks of the callus initiation. This is similar to a protocol of biolistic-mediated transformation of rice (Banakar and Wang, 2020). Most importantly, this practice helps to avoid the uncertainty of using one particular callus line in obtaining transgenic events.

To ensure germination and avoid contamination, removing seed casing and using proper disinfection procedure is critical. In our experiments, any efforts to germinate teosinte seeds *in vitro* with intact seed casing were unsuccessful, either due to contamination or non-synchronous germination. Unlike maize, fresh teosinte seeds undergo a period of seed dormancy after harvesting. Lopez et al. (2011) determined the strength of seed dormancy by measuring the length of time from harvest until the seeds achieving >80% germination. Six levels of seed dormancy were categorized by Lopez et al. (2011), ranging from no dormancy (Level 0, seeds germinate immediately after the drying process) to very deep dormancy (Level 5, seeds germination reach only 50% 1year after harvesting). The teosinte seeds used in this work could reach 50% of germination 3months after harvest, likely fall into weak dormancy (Level 1) according to Lopez et al. (2011). To accelerate progeny analysis, we treated the freshly harvested T1 seeds with GA3 to break the dormancy (Mondrus-Engle, 1981), removed the hard casing, and germinated them in ½ MS+GA3 germination medium. This proved to be successful as we were able to achieve ~25% germination rate 2weeks after the GA3 treatment, obtaining some T1 seedlings needed for progeny analysis. Compared to seeds harvested in the field, seeds harvested from the growth chamber grown plants were relatively clean, required no sterilization treatment before being placed on the germination medium. To prevent contamination, it can also be considered to include anti-fungal or antibiotics into the germination media if necessary.

To our knowledge, this is the first report describing a robust protocol for establishing embryogenic callus culture from mature seed-derived leaf segment and regenerating fertile plants in teosinte, *Zea parviglumis*. Embryogenic callus culture production and regeneration have been reported in *Zea diploperennis* some 35years ago (Prioli et al., 1984; Sondahl et al., 1984; Swedlund and Locy, 1988). Auxins have been proven important in teosinte callus initiation and regeneration. All previous work on teosinte regeneration included 2,4-D in their culture media (Prioli et al., 1984; Sondahl et al., 1984; Swedlund and Locy, 1988). In our callus induction media, in addition to 2,4-D, auxins picloram (for MSW57) or dicamba (for 605B) was also included.

Multiple factors play important roles collectively for the success of our teosinte regeneration protocol. One major difference in our protocol compared to the previous publications is that we used explants generated from *in vitro* germinated teosinte seeds, instead of field grown seedling materials (Prioli et al., 1984; Sondahl et al., 1984; Swedlund and Locy, 1988). This practice allowed us to germinate seeds in artificial culture media containing growth hormones. Inspired by callus induction in seedlings derived from maize mature seeds (Sidorov et al., 2006), we compared callus production and regeneration frequencies from seeds germinated in ½ MS medium and MSVS34 which contains picloram and BAP. The explants collected from the growth hormones-primed seeds (i.e., MSVS34-P2.2) produced similar rates of callus culture as compared to that of from hormone-free medium (i.e., ½ MS) but resulted in much higher frequencies for fertile regenerants (Table 1).

Another important step in our teosinte regeneration protocol was the addition of a maturation step, in which callus pieces were subcultured onto medium supplemented with both BAP and IAA before moved to rooting medium. Without the maturation step, very few plants could be regenerated (Supplementary Figure S3B).

Using the callus induction and regeneration protocol described in this work, we could maintain the teosinte callus lines for at least 15months and regenerate fertile plants, although their abilities to regenerate diminished over time. In general, the transformation experiments should be performed using younger callus tissue as they are more vigorous in growth and regeneration. In our bombardment experiments, both 3-month-old and 1-month-old callus cultures produced transgenic events (Table 1).

Overall, the frequency of transformation was low (4.17%) which means there is more room for improvement. While we conducted comparison on effect of growth hormones on regeneration, our experimental designs were in broad strokes. Further experiments to optimize the growth media used in teosinte transformation have the potential to greatly enhance transformation frequency. For example, the two media (½ MS vs. MSVS34-P2.2) used for germination contained many different components other than auxins. Therefore, further detailed, and refined experiments will be needed to determine which components contribute most to the regeneration improvement. Additionally, there are many parameters and numerous variables in the bombardment process that could further be evaluated and improved.

The successful transformation of teosinte is an important advance in studying this proposed progenitor of modern maize. While the transformation frequency remains low, it is possible for researchers to introduce CRISPR reagents to teosinte cells for genome editing. We are now in a position to interrogate the teosinte genome and deepen our understanding for its role in maize domestication.

## Data Availability Statement

The original contributions presented in the study are included in the article/Supplementary Material, and further inquiries can be directed to the corresponding author.

## Author Contributions

KW initiated and oversaw the entire project. JZ conducted experiments of callus regeneration improvement and performed transformation using pKL2155, plant care, and PCR analysis. SM-O initiated experiments of callus induction and regeneration and performed transformation using pAHC25, plant care, and Southern blot hybridization analysis. KL designed and built the construct pKL2155 and performed qPCR analysis. MA performed progeny phenotyping. QJ performed Southern blot hybridization analysis. JZ, KL, and KW performed data analysis and prepared the manuscript. All authors contributed to the article and approved the submitted version.

## Funding

This project was partially supported by National Science Foundation Plant Genome Research Program Grants 1917138 to KW, by Predictive Plant Phenomics Research Traineeship Program (National Science Foundation Grant DGE-1545453) to JZ, by the USDA NIFA Hatch project #IOW04341, and by State of Iowa funds, and by the Crop Bioengineering Center of Iowa State University.

## Conflict of Interest

The authors declare that the research was conducted in the absence of any commercial or financial relationships that could be construed as a potential conflict of interest.

## Publisher’s Note

All claims expressed in this article are solely those of the authors and do not necessarily represent those of their affiliated organizations, or those of the publisher, the editors and the reviewers. Any product that may be evaluated in this article, or claim that may be made by its manufacturer, is not guaranteed or endorsed by the publisher.

## Footnotes

^1^ https://www.ars-grin.gov/

^2^ https://www.restorationseeds.com/products/teosinte

^3^ https://www.socscistatistics.com/tests/ztest/default2.aspx
