# Supplementary material for: Transformation of Teosinte (Zea mays ssp. parviglumis) via Biolistic Bombardment of Seedling-Derived Callus Tissues
Source: Front Plant Sci. 2021 Dec 9;12:773419. doi: 10.3389/fpls.2021.773419 (PMC8696365; doi:10.3389/fpls.2021.773419)
Supplement: Supplementary file 1 [file Data_Sheet_1.pdf]

Supplementary Materials

Transformation of teosinte (*Zea mays* ssp. *parviglumis*) via biolistic bombardment of leaf derived callus tissues

Jacob D. Zobrist[1,2,3], Susana Martin-Ortigosa[1,^], Keunsub Lee[1,2], Mercy K. Azanu[1,2,4], Kan Wang[1,2]*

[1]Department of Agronomy, Iowa State University, Ames, Iowa, USA
[2]Crop Bioengineering Center, Iowa State University, Ames, Iowa, USA
[3]Interdepartmental Genetics and Genomics Major, Iowa State University, Ames, Iowa, USA
[4]Interdepartmental Plant Biology Major, Iowa State University, Ames, Iowa, USA

^Current address: Plant Cell & Transformation Technologies, Research & Development, KWS SAAT SE & Co. KGaA, Einbeck, Germany

*Corresponding Author: Kan Wang, kanwang@iastate.edu

Keywords:

embryogenic callus, gene gun, genetic transformation, growth media, herbicide resistance, mature seed, *Zea parviglumis*

Running title:
Biolistic-mediated teosinte transformation

Table S1. Media components

Purpose	Medium name	Reference	Ingredient
Seed sterilization			50% commercial bleach (8.25% sodium hypochlorite), 0.1% Tween® 20.
Germination	½ MS	Murashige and Skoog (1962)	2.2 g/L MS basal medium, 20 g/L sucrose, pH 5.6, 5 g/L agar; autoclave; cool down; add antibiotics meropenem (10 mg/L) and/or fungicide benomyl (50 mg/L) if needed. Pour medium into 25 x 100 mm Petri dishes.
Germination	MSVS34-P2.2	Sidorov et al (2006)	4.4 g/L MS basal medium, 40 g/L maltose, 0.1 g/L casein hydrolysate, 0.75 g/L magnesium chloride, 0.5 g/L glutamine, 1.95 g/L 2-ethanesulfonic acid (MES), pH 5.6, 6 g/L agar; autoclave; cool down; add 3 mg/L 6-benzylaminopurine (BAP), 0.1 g/L ascorbic acid, 2.2 mg/L picloram; add antibiotics meropenem (10 mg/L) and/or fungicide benomyl (50 mg/L) if needed. Pour medium into 25 x 100 mm Petri dishes.
Callus induction	MSW57	Sidorov et al (2006)	4.4 g/L MS basal medium, 30 g/L sucrose, 0.5 g/L casamino acid, 0.5 mg/L thiamine HCL, 1.38 g/L L-proline, 0.5 mg/L 2,4-dichlorophenoxyacetic acid (2,4-D), pH 5.6, 6 g/L agar; autoclave; cool down; add 2.2 mg/L picloram, 3.4 mg/L silver nitrate, 10 mg/L meropenem. Pour medium into 20 x 100 mm Petri dishes.
Callus induction	605B	Masters et al (2020)	4.3 g/L MS basal salt mixture, 0.6x N6 macro salts[1], 0.6x B5 minor salts[2], 0.4x Eriksson's vitamins[3], 0.6x S&H vitamins[4], 0.6x ferrous sodium stock[5], 1.68 g/L potassium nitrate, 0.2 mg/L thiamine HCl, 0.3 g/L casein hydrolysate, 2 g/L proline, 20 g/L sucrose, 0.6 g/L glucose, 0.8 mg/L 2,4-D, pH 5.6, 6 g/L agar; autoclave; cool down; add 1.2 mg/L dicamba, 3.4 mg/L silver nitrate, 10 mg/L meropenem. Pour medium into 20 x 100 mm Petri dishes.
Osmotic treatment for bombardment	MSW57 osmotic	Sidorov et al (2006)	4.4 g/L MS basal medium, 30 g/L sucrose, 0.5 mg/L casamino acid, 0.5 mg/L thiamine, 1.38 g/L L-proline, 0.5 mg/L 2,4-dichlorophenoxyacetic acid (2,4-D), 36.4 g/L sorbitol, 36.4 g/L mannitol, pH 5.6, 6 g/L agar; autoclave; cool down; add 2.2 mg/L picloram, 3.4 mg/L silver nitrate, 10 mg/L meropenem. Pour medium into 60 x 15 mm Petri dishes.
Maturation	13329iaa	Masters et al (2020)	4.3 g/L MS basal salt mixture, 5 mL/L 200x MS vitamins home-made mix[6], 60 g/L sucrose, 0.1 g/L myo-inositol, 0.7 g/L L-proline, 1.25 mg/L $CuSO_4$, 0.5 mg/L zeatin, pH 5.6, 8 g/L agar; autoclave; cool down; add 1 mg/L BAP, 1 mg/L indoleacetic acid (IAA), 10 mg/L meropenem. Pour medium into 20 x 100 mm Petri dishes.
Rooting	272iba	Lowe et al (2016)	4.3 g/L MS basal salt mixture, 5 mL/L 200x MS vitamins home-made mix[6], 40 g/L sucrose, 0.1 g/L myo-inositol, pH 5.6, 6 g/L agar; autoclave; cool down; add 0.5 mg/L indole-3-butyric acid (IBA), 10 mg/L meropenem. Pour medium into 25 x 100 mm Petri dishes.

1. N6 Macro Nutrient Stock (10x): $CaCl_2 \cdot 2H_2O$, 1.66 g/L; $(NH_4)_2SO_4$, 4.62 g/L; KH_2PO_4, 4 g/L; $MgSO_4 \cdot 7H_2O$, 1.85 g/L; KNO_3, 28.3 g/L.
2. B5 Micro Nutrient Stock (1000x): Boric Acid, 3 g/L; $MnSO_4 \cdot H_2O$, 10 g/L; $Na_2MoO_4 \cdot 2H_2O$, 0.25 g/L; KI, 0.75 g/L.
3. Eriksson's vitamins (1000x in liquid): Phytotech E330; Eriksson, 1965
4. Schenk and Hildebrandt vitamins (100x in powder): Phytotech S826; Schenk and Hildebrandt, 1972
5. Ferrous sodium stock (100x): EDTA-$Na_2 \cdot 2H_2O$, 3.7 g/L; $FeSO_4 \cdot 7H_2O$ 2.79 g/L.
6. MS vitamin home-made mix (200x): 0.1 g/L nicotinic acid, 0.02 g/L thiamine HCL, 0.1 g/L pyridoxine·HCL, 0.4 g/L glycine

Table S2a. Primer pairs and plasmid DNA used for making construct pKL2155

Primer name	Primer sequence	Plasmid template (reference)	Tm (°C)	Amplicon size (kb)
ALS1_F1	CCGCCAGATCTTCCGGATGGCTCGAGTTTTTCAGCAAGATGCCCTTACTTTAACGCCTCT	PHP81814 (Chu et al., 2019)	55.5	1.2
ALS1_R1	ACCTGCACATCAACAAATTTTGGTCATATATTAGAAAAGTAAACTACAGAAAAGCAATTG			
ALS2_F1	ACTTTTCTAATATATGACCA	PHP81814 (Chu et al., 2019)	52.1	2.7
ALS2_R1	CATTTTTGGAGTAGGGGTAGGGAATACTAAGGGCCCAAGGTATGTTGCTTAGGCCTCTTT			
P35S_F1	CCTTGGGCCCTTAGTATTCC	pKL2013 (McCaw et al., 2021)	62.8	0.8
P35S_R1	CCTCGCCCTTGCTCACCATGACCTTTCTCTTCTTCTTTGGGGATCCCCCGGGACCGGTCC			
tdTomato_F1	CCAAAGAAGAAGAGAAAGGT	pGreenIIM DR5v2-ntdTomato/DR5-n3GFP (Liao et al., 2015)	58.3	1.7
tdTomato_R1	ATGGCAGCTGAGAATATTGTAGGAGATCTTCTAGAAAGATACATAGATGACACCGCGCGC			

Table S2b. Primers and probes used for transgene copy number estimation by qPCR

Primer name	Primer sequence	Template (reference)	Tm (°C)	Amplicon size (bp)
ZvMEK1-F1	CTCGAGTTTTTCAGCAAGATCTCAACTGCAGCCTCCCTAC	*Z. parviglumis* genomic DNA (This study and Hufford et al., 2021)	68	281
ZvMEK1-R1	GGGGTGAAATAGCGTCCTTGA			
GUS-F2	TCAAGGACGCTATTTCACCCCTGCTGTGCCTGAACCGTTAT	pAHC25 (Christensen & Quail, 1996)	68	284
GUS-R2	CCATACCTGTTCACCGACGA			
PSbAls-F1	TCGTCGGTGAACAGGTATGGGGTGTCAGTGCCGTTTACTC	pKL2155 (This study)	67	339
PSbAls-R1	AGGAGATCTTCTAGAAAGATTGTGTGGCTCAGATGGGAAT			
ZvMEK1-qPCR-F1	TCCGTGTATGGTCACCTTATTG	pKL2347* and *Z. parviglumis* genomic DNA (This study)	60	96
ZvMEK1-qPCR-R1	TGAATCCACCGCTGATAAGAC			
ZvMEK1-PROBE1	TATTGTTGCGACGGGCCTTGTGAT			
GUS-qPCR-F1	GAATACGGCGTGGATACGTTAG	pKL2347* and *Z. parviglumis* genomic DNA (This study)	60	106
GUS-qPCR-R1	GATCAAAGACGCGGTGATACA			
GUS-PROBE1	TGAAGAGTATCAGTGTGCATGGCTGG			
PSbAls-qPCR-F1	CGTGTCAGTACCGTCTGTAGAT	pKL2347* and *Z. parviglumis* genomic DNA (This study)	60	132
PSbAls-qPCR-R1	GCTGGTGCGTCAAGAAGATT			
PSbAls-Probe 1	CCGTCGTCCACAGTCATGTCCAAA			

* pKL2347 is a 3797 bp plasmid containing DNA fragments of the reference gene *ZvMEK1* (261 bp), *gus* gene (263 bp) and *SbAls* promoter (299 bp). This plasmid was used for generating standard curves for qPCR.

Table S3a. Primer pairs used for PCR analysis

Primer name	Forward primer	Reverse primer	Product size (bp)	Coordinates
GUS	GGGCCAACTCCTACCGTACC	AGTGCGCTTGCTGAGTTTCC	201	*gus* gene<br>2292-3192 on pAHC25
Bar	ATCGAGACAAGCACGGTCAA	TCAGCAGGTGGGTGTAGAGC	250	*bar* gene<br>6239-6488 on pAHC25
Primer 1<br>(F1R1)	GACAACAACATGGCCGTCA	CTTGTACAGCTCGTCCATGC	696	*tdTomato* gene<br>5557-6252 on pKL2155
Primer 2<br>(F2R2)	GCACAATCCCACCCCTACT	CCATGTTGTTGTCCTCGGAG	1243	P35S to *tdTomato*<br>4327-5569 on pKL2155
Primer 3<br>(F3R3)	ACTTGAGGAAGTCCGATGGC	ACTGACACCGACCAGGGAT	201	*SbAls* Promoter<br>283-483 on pKL2155<br>(provided by Frank McFarland, U Wisconsin)

Table S3b. PCR conditions

STEP	GUS/bar	tdTomato	35S-tdTomator	SbALS
Initial Denaturation	98°C/30 s	98°C/30 s	98°C/30 s	98°C/30 s
Denature	98°C/10 s	98°C/10 s	98°C/10 s	98°C/10 s
Annealing	64°C/30 s	66°C/30 s	66°C/30 s	67°C/30 s
Extension	72°C/60 s	72°C/60 s	72°C/75 s	72°C/15 s
Final Extension	72°C/5 min	72°C/5 min	72°C/5 min	72°C/5 min

Table S3c. PCR analysis on T0 teosinte events transformed with pKL2155

Sample ID	Total regenerated plants	# plants moved to soil and PCR analyzed	# plants tested positive for at least one primer pair	# positive for PCR primer 1 (696 bp)	# positive for PCR primer 2 (1243 bp)	# positive for PCR primer 3 (201 bp)
**Event 1**	36	16	16	14	16	14
**Event 2**	6	5	5	5	2	2
**Event 3**	2	2	2	2	2	2
**Event 4**	1	1	1	1	1	1
**Event 5**	4	3	3	3	3	3
**Total**	**49**	**27**	**27**	**25**	**24**	**22**

Table S4a. Partial data from Table 1

Trtmnt	Germination media	Callus induction media	Total # whorl segment	# Whorl segment regenerated	Plant regeneration rate*
1	1/2 MS	MSW57	11	0	0.0%[a]
2		605B	11	2	18.2%[ab]
3	MSVS34-P2.2	MSW57	10	5	50.0%[bc]
4		605B	10	7	70.0%[c]

*Regeneration rates with same letters are not statistically significantly different.

Table S4b. Statistical analysis of different germination and callus induction media*

Treatment comparison	Values		Significance	
	z	*p*	at $p < 0.05$	at $p < 0.01$
1 vs 2	-1.4832	0.13888	ns	-
1 vs 3	-2.6868	0.00714	s	s
1 vs 4	-3.3985	0.0007	s	s
2 vs 3	-1.5448	0.12356	ns	-
2 vs 4	-2.3965	0.0164	s	ns
3 vs 4	-0.9129	0.36282	ns	-

*Two-tailed *Z*-test of difference in proportion (https://www.socscistatistics.com/tests/ztest/default2.aspx)
ns, non-significant; s, significant

References:

Chu, U. C., Adelberg, J., Lowe, K., Jones, T. J. (2019). Use of DoE methodology to optimize the regeneration of high-quality, single-copy transgenic *Zea mays* L. (maize) plants. *In Vitro Cell.Dev.Biol.-Plant* 55, 678–694. https://doi.org/10.1007/s11627-019-10002-w.

Christensen, A. H., and Quail, P. H. (1996). Ubiquitin promoter-based vectors for high-level expression of selectable and/or screenable marker genes in monocotyledonous plants. *Transgenic Res*. 5, 213–218. doi:10.1007/BF01969712.

Eriksson T (1965) Studies on the growth requirements and growth measurements of cell cultures of Happlopapus gracilis. *Physiol Plant* 18, 976-993.

Hufford, M. B., Seetharam, A. S., Woodhouse, M. R., Chougule, K. M., Ou, S., Liu, J., et al. (2021). De novo assembly, annotation, and comparative analysis of 26 diverse maize genomes. *Science* 373, 655-662. doi: 10.1126/science.abg5289.

Liao, C.Y., Smet, W., Brunoud, G., Yoshida, S., Vernoux, T., Weijers, D. (2015). Reporters for sensitive and quantitative measurement of auxin response. *Nat. Methods* 12, 207-210. doi: 10.1038/nmeth.3279.

Lowe, K., Wu, E., Wang, N., Hoerster, G., Hastings, C., Cho, M-J., et al. (2016). Morphogenic regulators *Baby boom* and *Wuschel* improve monocot transformation. *Plant Cell* 28, 1998-2015. doi:10.1105/tpc.16.00124.

Masters, A., Kang, M., McCaw, M., Zobrist, J. D., Gordon-Kamm, W., Jones, T., Wang K (2020). *Agrobacterium*-mediated immature embryo transformation of recalcitrant maize inbred lines using morphogenic genes. *J. Vis. Exp.* 156, e60782, doi: 10.3791/60782.

McCaw, M.E., Lee, K., Kang, M., Zobrist, J.D., Azanu, M.K., Birchler, J.A., Wang, K. (2021). Development of a Transformable Fast-Flowering Mini-Maize as a Tool for Maize Gene Editing. *Front. Genome Ed*. 2: 622227. doi: 10.3389/fgeed.2020.622227.

Murashige, T., Skoog, F. (1962). A revised medium for rapid growth and bio assays with tobacco tissue cultures. *Physiol. Plant* 15, 473-97.

Schenk, R. U., Hildebrandt A. C. (1972). Medium and techniques for induction and growth of monocotyledonous and dicotyledonous plant cell cultures. *Can. J. Bot.* 50, 199-204.

Sidorov, V., Gilbertson, L., Addae, P., Duncan, D. (2006). *Agrobacterium*-mediated transformation of seedling-derived maize callus. *Plant Cell Rep.* 25, 320-328. doi:10.1007/s00299-005-0058-5.

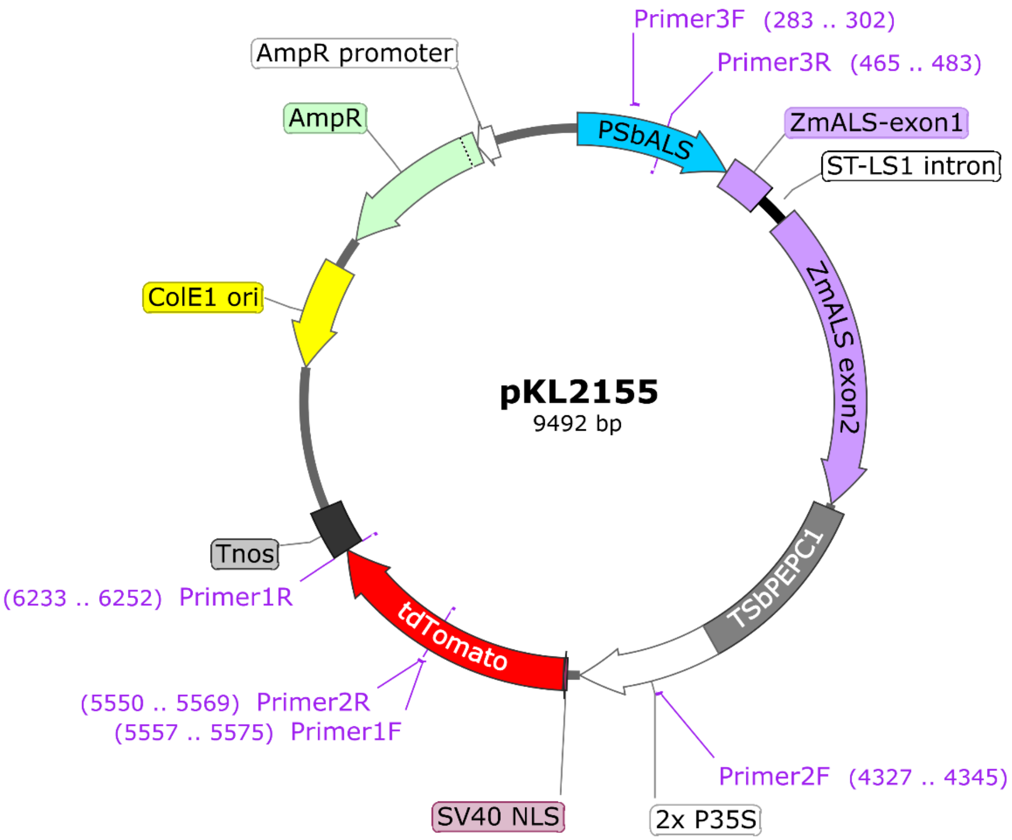

Figure S1. Map of the reporter plasmid pKL2155. The plasmid has genes for the mutant acetolactate synthase (ALS) from maize (*ZmAls*) for herbicide resistance selection and the red fluorescent protein tdTomato for visual screening. Expression of the *ZmAls* gene, which is separated by a potato ST-LS1 IV2 intron (ST-LS1), is driven by the sorghum *Als* promoter (P*SbAls*) and terminated by the sorghum *PepC* gene terminator (T*SbPepC1*). Expression of the *tdTomato* gene is driven by a 2x CaMV 35S promoter (2x P35S) and terminated by an *Agrobacterium* nopaline synthase terminator (T*nos*). An SV40 nuclear localization signal is included at the N-terminus of the tdTomato protein. ColE1 ori, high copy number origin of replication for *E. coli*. Amp^R, ampicillin resistance gene.

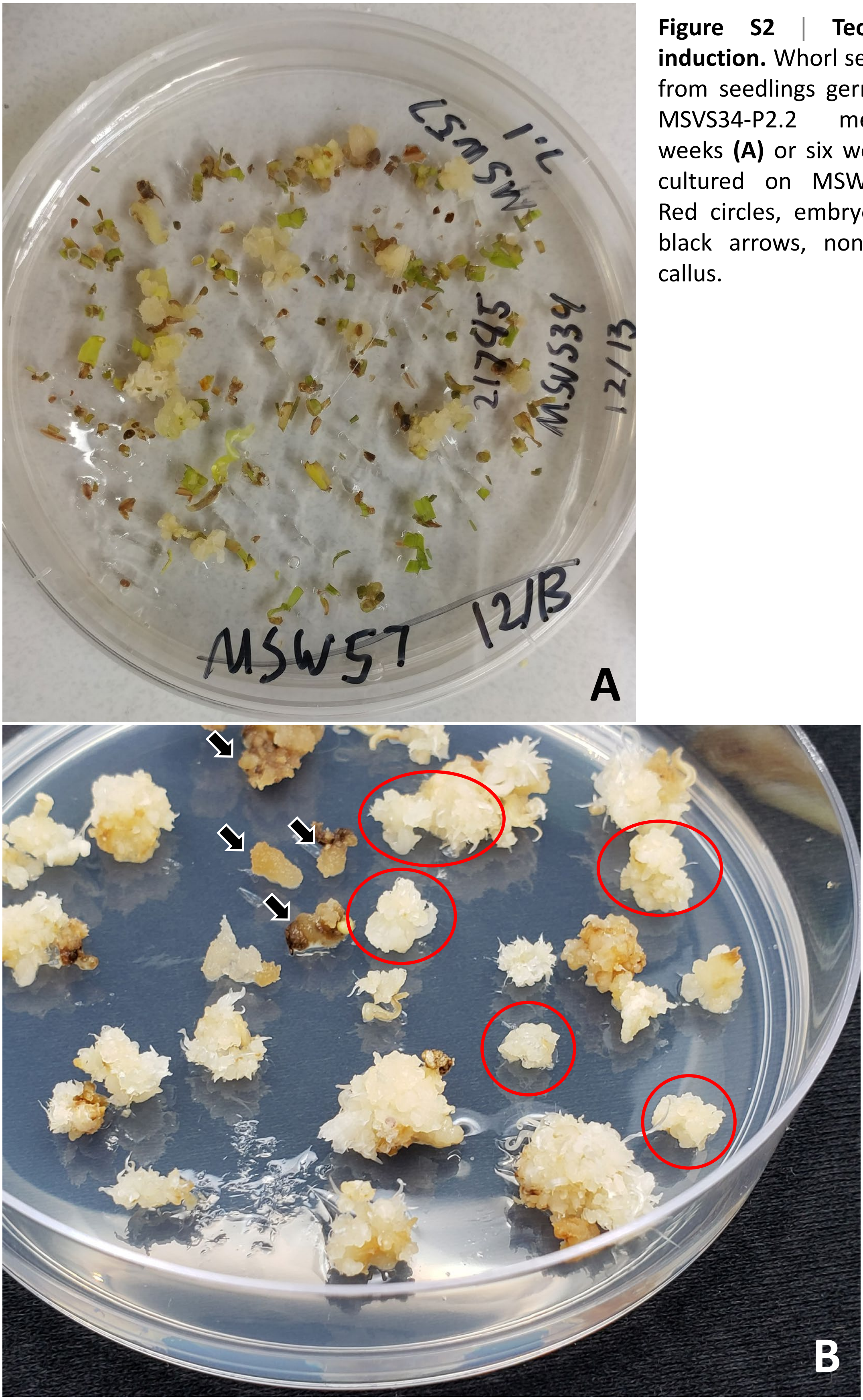

Figure S2 | Teosinte callus induction. Whorl segment pieces from seedlings germinated from MSVS34-P2.2 medium two weeks **(A)** or six weeks **(B)** after cultured on MSW57 medium. Red circles, embryogenic callus; black arrows, non-embryogenic callus.

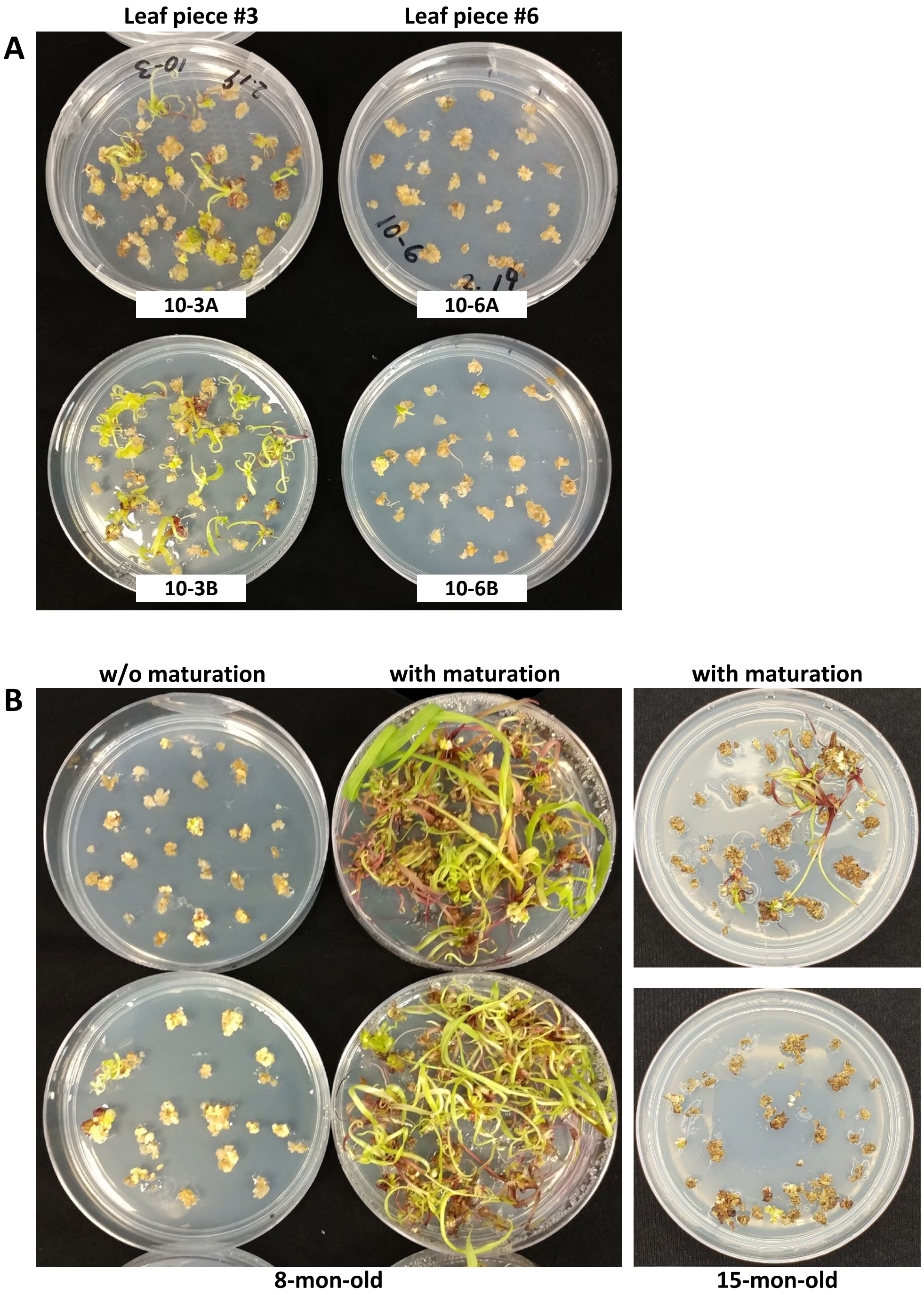

Figure S3 | **Comparison of callus regenerability. (A)** Two plates of callus culture from the leaf piece #3 (left) and #6 (right) generated from the same plant (28 days after placing on Maturation medium). This shows that different leaf pieces from same plant have different regenerability using the same medium. **(B)** Two plates of 8-mon-old callus line #10-3 regenerated without (left) and with (center) maturation step, and same callus line regenerated 15-mon (right) after the callus initiation with the maturation step. This shows that (1) including the maturation step is important for regeneration, and (2) the regenerability of callus line diminished after continuous cultivation.

A. Bialaphos kill curve

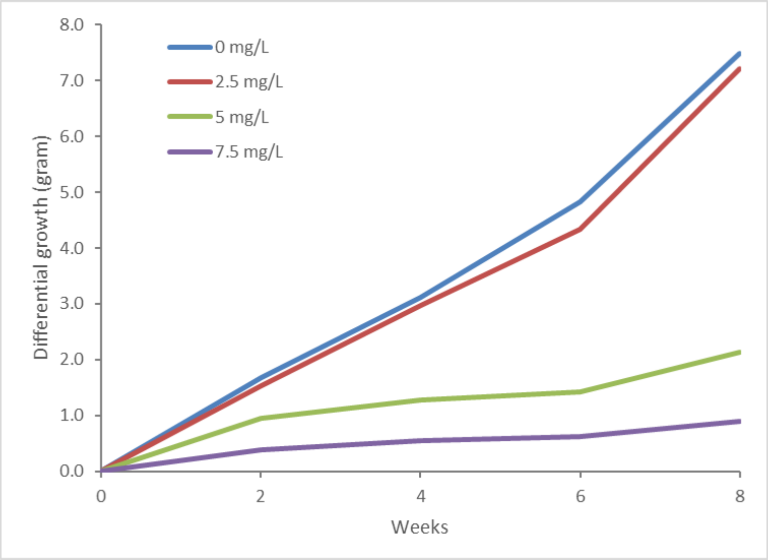

B. EMS kill curve (exp 1)

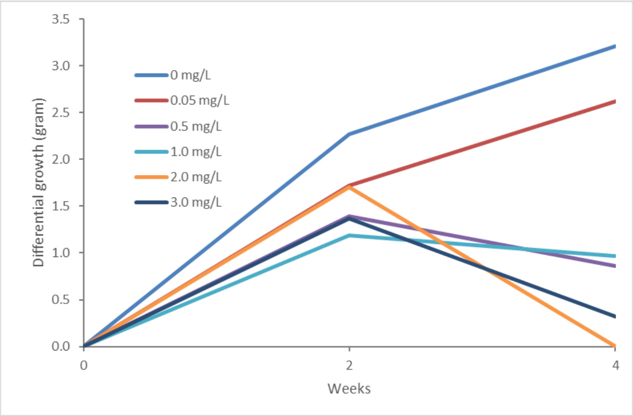

C. EMS kill curve (exp 2)

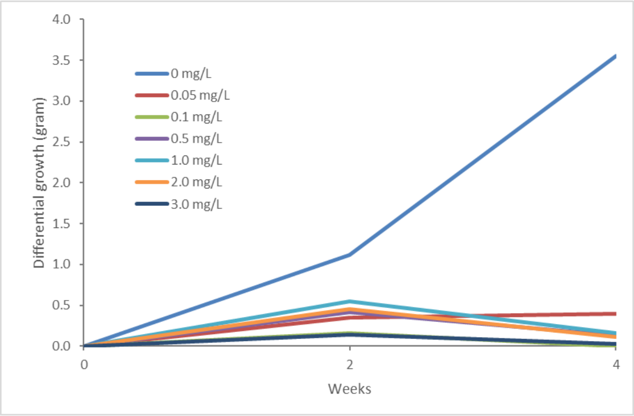

Figure S4 | Herbicide kill curves of teosinte callus cultures. (A) bialaphos; **(B,C)** ethametsulfuron (EMS).

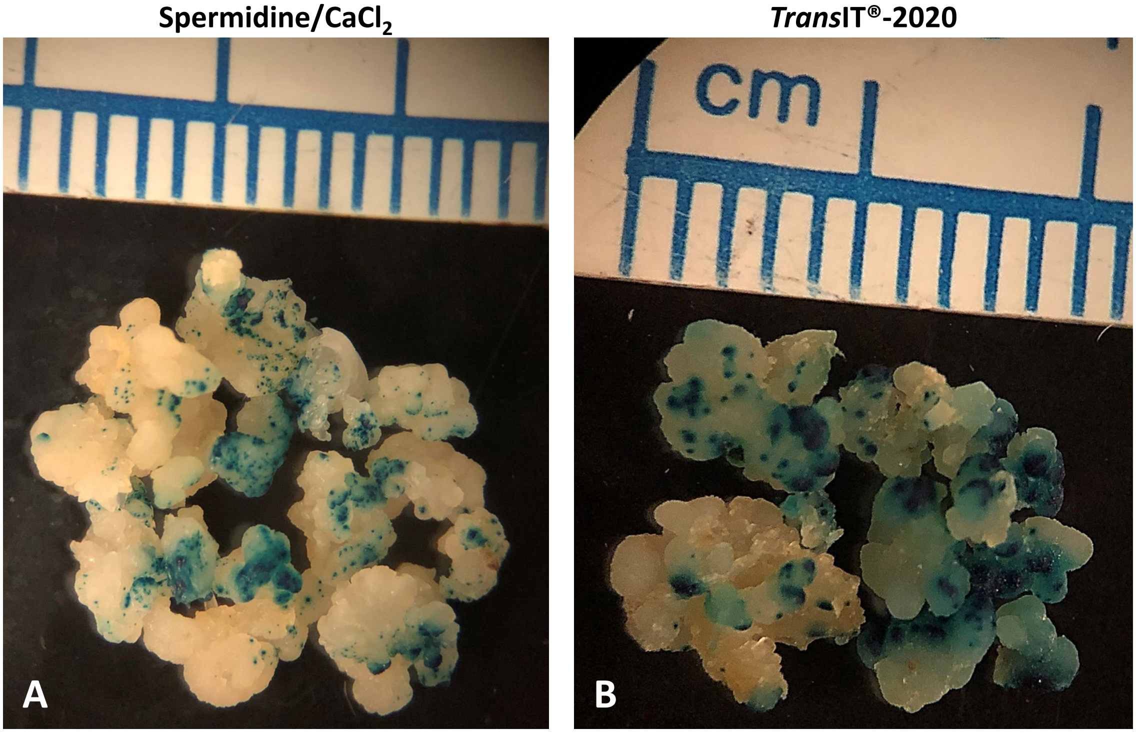

Figure S5 | **Comparison of DNA/gold coating agents on DNA delivery.** Transient GUS assay on bombarded teosinte callus using pAHC25 DNA/gold coated with spermidine/$CaCl_2$ **(A)** or *Trans*IT® -2020 **(B)**. Blue color indicates the *gus* gene expression.

A. Primer pairs #2 (F2R2)

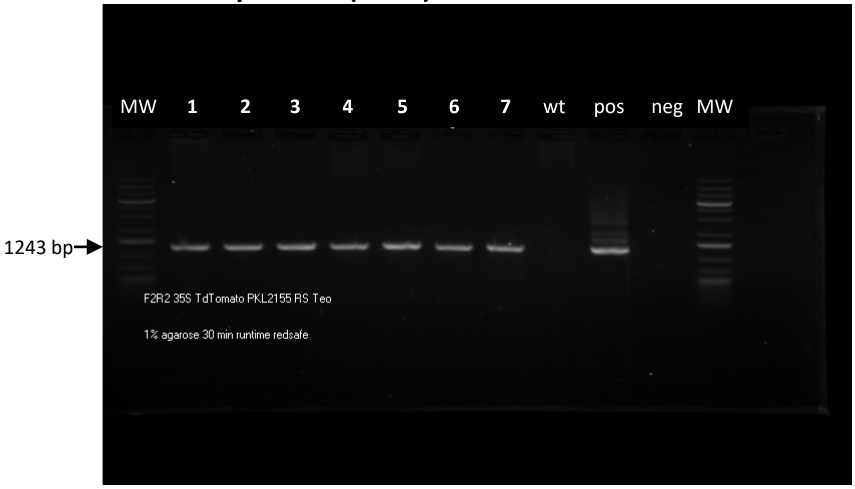

B. Primer pairs #3 (F3R3)

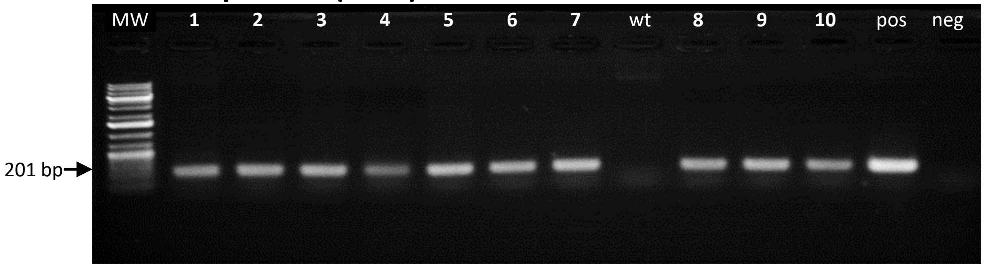

Figure S6 | PCR analysis of pKL2155 transgenic T0 plants. Representative PCR results from primer pairs #2 (F2R2) **(A)** and primer pairs #3 (F3R3) **(B)**. Samples #1 to #10 are selected individual plants regenerated from five independent callus events. Primer sequences and products information can be found in Tables S3a and S3b. MW, 1 kb-ladder; wt, wild type teosinte; pos, plasmid pKL2155 as positive control; neg, water as negative control.

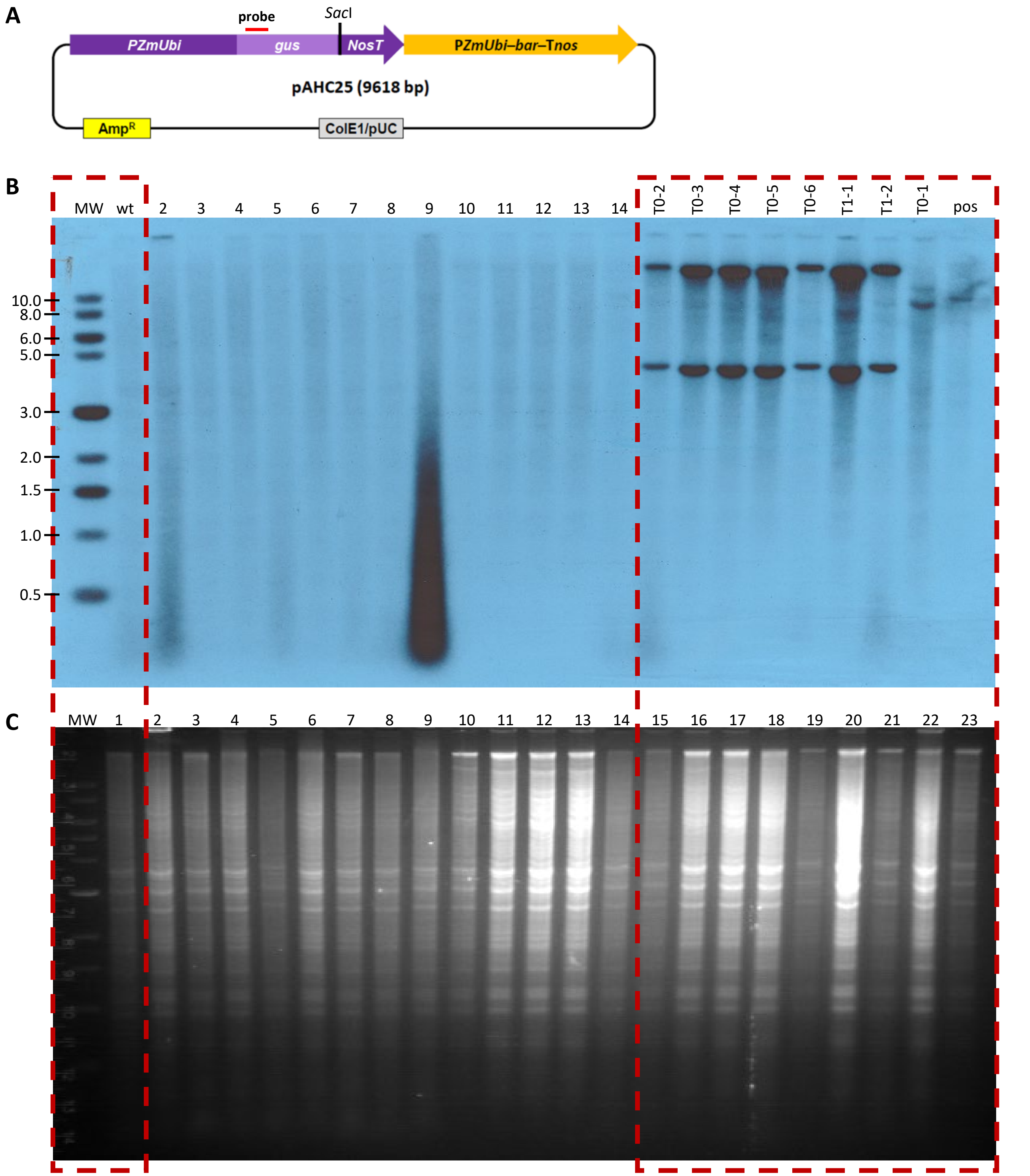

Figure S7 | Southern blot hybridization analysis of pAHC25 transgenic plants. (A) Scheme of pACH25 plasmid. Probe, 421 bp PCR fragment amplified from the *gus* gene. **(B)** Complete image of the Southern blot. Fifteen micrograms of genomic DNA were digested with *Sac*I restriction enzyme, which cuts only once in the plasmid. The membrane was hybridized with ^{32}P-labeld *gus* probe. MW, 2-Log DNA Ladder; wt, wild type teosinte; Lanes 2-14, candidate plants derived from one bialaphos resistant event; these plants appeared to contain no transgene; T0-2 to T0-6, transgenic T0 plants derived from another bialaphos resistant event (pAHC25-1), showing two major bands hybridizing with the *gus* probe; T1-1 and T1-2, T1 plants of pAHC25-1 event; T0-1, a regenerated plant derived from pAHC25-1 that plant did not survive; pos, wild type teosinte genomic DNA spiked with pACH25 plasmid. **(C)** Agarose gel showing the genomic DNA digestion with SacI restriction enzyme before being blotted.
